# Decoding cell–cell communication in spatial transcriptomics: mechanistic insights, modeling constraints, and analytical caveats

**DOI:** 10.1093/bib/bbag477

**Published:** 2026-09-07

**Authors:** Yuesong Wu, Haohao Su, Yuehua Cui

**Affiliations:** Department of Statistics and Probability, Michigan State University, 619 Red Cedar Road, East Lansing 48824, MI, United States; Department of Statistics and Probability, Michigan State University, 619 Red Cedar Road, East Lansing 48824, MI, United States; Department of Statistics and Probability, Michigan State University, 619 Red Cedar Road, East Lansing 48824, MI, United States

**Keywords:** cell–cell communication, spatial transcriptomics, ligand–receptor interactions, tissue heterogeneity, computational methods

## Abstract

Cell–cell communication (CCC) is essential for maintaining tissue organization and driving biological progression, yet its inference from transcriptomic data has long been limited by the absence of spatial context. Advances in spatial transcriptomics (ST) now enable mechanistically grounded analyses of CCC by preserving the physical organization of cells and their microenvironments. In this review, we examine recent methodological developments in CCC inference from ST data, focusing on how statistical, optimal transport, and deep learning frameworks incorporate spatial information to model ligand–receptor (LR) interactions and downstream signaling. We also summarize key mechanism-driven components shared across spatial and non-spatial CCC approaches. In addition, we discuss how tissue heterogeneity and spatial architecture can introduce context-dependent biases, particularly for permutation-based inference, and outline mechanistic considerations such as LR biochemistry, signal transduction, and condition-specific communication. We further highlight databases that curate intercellular conduction and intracellular signaling processes. By integrating spatial constraints with biochemical and computational principles, this review offers an integrated assessment of the opportunities and limitations of current approaches. We conclude by identifying key methodological challenges and future directions for developing robust, scalable, and mechanistically interpretable CCC inference as ST technologies continue to advance.

## Introduction

Cell–cell interaction (CCI) and communication (CCC) play fundamental roles in maintaining the organization and function of multicellular organisms and underlie critical cellular decisions such as development, cell growth and division, differentiation, migration, and apoptosis [1]. We conceptualize CCIs as an umbrella term encompassing all intercellular information-exchange mechanisms, including both chemical signaling [e.g. ligand–receptor (LR) interactions [2]] and non-chemical intercellular communication mechanisms such as mechanical coupling [3], electrical conduction [4], and direct cytoplasmic continuity [5, 6]. Within this framework, we refer to CCC as the chemical mode of intercellular interaction that encompasses both intercellular LR conduction and intracellular downstream signal transduction [7]. Specifically, we refer to signaling genes as those related to LR-related protein–protein interaction (PPI) cascades [8], transcription factor (TF) activation [9], and target gene regulation [10].

**Figure 1:**
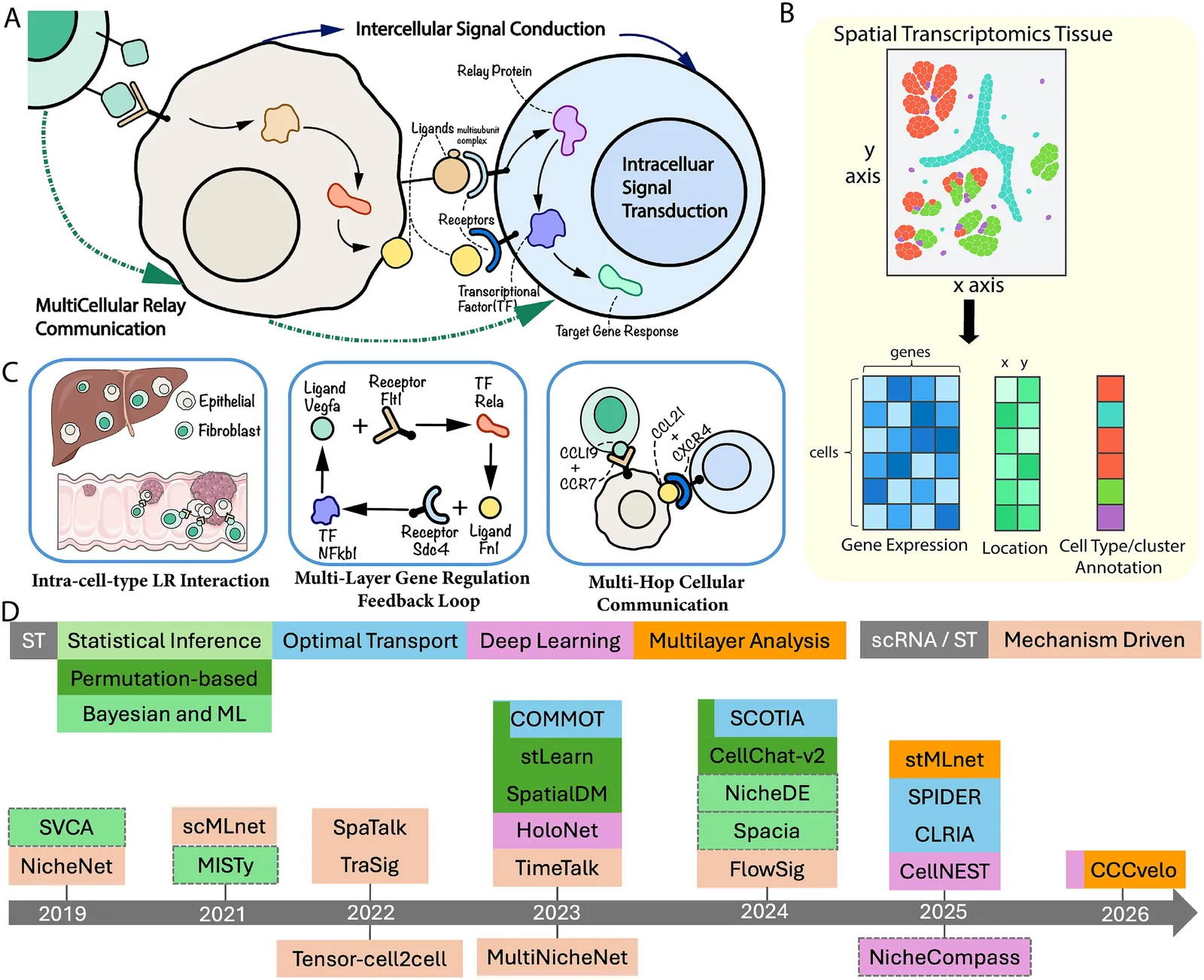
CCC and spatial transcriptomics data. (A) Schematic illustration of CCC. Intercellular signal conduction refers to ligand diffusion and LR interactions. Intracellular signal transduction refers to molecular relay processes within the receiver cell, including signaling cascades, TF activation, and target gene responses. Multicellular relay communication describes the sequential propagation of signals across chains of cells. (B) Spatial transcriptomics data measure gene expression together with cell or spot spatial locations, with additional cell-type or cluster annotations. (C) Illustration of intra-cell-type LR interaction, multi-layer regulation feedback loop and multi-hop cellular communication. Icons were obtained from Bioicons, the colon-cancer icon is by Servier and licensed under CC BY 3.0, and the healthy-liver icon is licensed under CC0. (D) Timeline of reviewed CCC methods. Approaches developed using ST data are categorized into statistical inference, optimal transport, deep learning and multilayer analysis frameworks . Methods that do not explicitly incorporate spatial location but nevertheless model key CCC mechanisms are categorized as mechanism driven methods. Methods indicated with dashed lines were originally developed for CCI analysis but can be readily adapted for CCC analysis.

Cells communicate through direct contact or over a distance through LR interactions [11] (Fig. 1A). In these processes, signaling ligands released by one cell bind to specific receptor complexes on another cell or, in some cases, on the same cell. Four major modes of ligand-mediated cell-to-cell communication can be distinguished: autocrine (self-communication), juxtacrine (direct contact between adjacent cells), paracrine (communication between nearby cells through ligand diffusion), and endocrine (long-distance communication through hormones traveling in the bloodstream) [12]. Additionally, neural or synaptic signaling represents a specialized form of long-distance communication, in which an electrical signal travels along the axon and triggers local LR interactions at the synapse [13, 14].

Recent technological advances, particularly next-generation sequencing and spatially resolved omics, have enabled high-resolution profiling of gene expression at the cellular level, providing a powerful foundation for deciphering CCC mechanisms [15]. Early CCC modeling approaches were developed using bulk RNA-seq [16] data by associating LR activity across samples [17, 18], and later evolved with single-cell RNA sequencing (scRNA-seq) [19] to resolve communication between defined cell types [20, 21]. However, both bulk and single-cell transcriptomic modalities lack spatial information and, therefore, overlook the biophysical constraints of LR interactions, particularly juxtacrine and paracrine signaling, which occur only over short distances. The advent of spatial transcriptomics (ST) [22] overcomes this limitation by preserving the spatial location of cells (Fig. 1B), making it fundamentally better suited for dissecting CCC mechanisms.

Several reviews have summarized the rapidly expanding field of CCC analysis. Su *et al*. [11] provide a comprehensive overview of cellular communication mechanisms, clinical relevance, and computational methods based on diverse technologies, while Cesaro *et al*. [23] and Armingol *et al*. [24] focus on recent advances using both scRNA-seq and ST technologies. These reviews thoroughly discuss how technological progress has shaped CCC analysis; however, they treat spatial CCC methods primarily as a subsection rather than a central theme. Santvoort and Eduati [25] take an algorithmic perspective, summarizing computational frameworks designed for deciphering CCI from ST data. This work does not explicitly distinguish between CCC and CCI methods and primarily emphasizes spatial distance as a local constraint to approximate juxtacrine and paracrine signaling, while overlooking the global role of spatial information in ST data, namely, its potential to account for tissue heterogeneity and model CCC in a spatially contextualized manner. For instance, Arora *et al*. [26] reported stronger communication between myofibroblastic cells and tumor cells in tumor-leading-edge subregions compared with tumor-core subregions, highlighting the influence of spatial heterogeneity and intra-cell-type discrepancy on intercellular communication.

Therefore, in this review, we focus on CCC methods (Fig. 1D) that leverage ST data and discuss how spatial information informs model design and how computational frameworks align with underlying biological mechanisms. In Section Spatial information matters in cell–cell communication inference, we review 16 CCC methods developed for ST data, with an emphasis on how spatial information is incorporated in the computational framework. In Section Mechanism-aware cell–cell communication beyond spatial proximity, we summarize eight additional mechanism-driven CCC inference methods, including those addressing non-spatial but biologically important biochemical mechanisms, functionally driven communication, and differential or shared communication patterns across multiple conditions. In Section Databases for cell–cell communication analysis, we briefly summarize available database related to both intercellular conduction and intracellular signal transduction.

## Spatial information matters in cell–cell communication inference

Early CCC methods developed for bulk RNA-seq and scRNA-seq data typically rely on averaging cellular communication signals at the cell-type level due to the lack of spatial information. While such aggregation improves robustness against sequencing dropouts in individual cells, it can introduce bias by conflating signals from spatially distant cells that are unlikely to participate in direct LR interactions [27]. Therefore, a key advantage of ST data is its ability to retain cell location information, thereby enabling the modeling of more biologically realistic CCC activity.

In early CCC inference methods, spatial information is usually used as local constraints for LR interactions to recapitulate realistic short-range LR binding mechanisms. However, beyond spatial proximity, ST data also enable the identification of heterogeneous tissue subregions [28–31], such that a tissue slice may contain tumor core, tumor edge, and tumor adjacent normal regions. Thereby, recent methods utilize this attribute of ST data to study region- or microenvironment-specific CCC patterns. Moreover, new methods also utilize the more realistic spatial constraint LR interactions to infer downstream CCC dysregulation, with the combination of intracellular signal transduction and gene-gene regulatory network.

In this section, we classify CCC methods that leverage ST data into four categories: (i) statistical inference-based methods, (ii) optimal transport (OT)-based mathematical frameworks, (iii) graph neural network (GNN)-based deep learning, and (iv) spatially induced multilayer signaling analysis approaches. We further discuss how each category incorporates spatial information as a local constraint, as well as a special case of long-distance cellular communication observed in neuronal data from the brain. Besides spatial constraints on LR interactions, multilayer signaling analysis-based algorithms further explicitly incorporate intracellular signal transduction into the model. In addition, we examine which approaches explicitly account for tissue heterogeneity and highlight key caveats associated with permutation-based inference and learning algorithms. A categorized overview of representative CCC methods discussed in this section, including their computational paradigms, use of spatial information, and inference strategies, is provided in Table 1.

**Table 1:** Summary of CCC methods, data modality, inference framework, cell-type handling, database usage, and spatial information usage

Method	Category	Data modality	Inference framework	Spot–cell type	Database	Spatial Information Usage
CellChatV3	CCC	ST	Permutation test	Weighted by cell-type deconvolution proportion	In-house CellChatDB	LR constraint: spatial weight *1/d*
stLearn	CCC	ST	Permutation test	Uses dominant cell type	CellPhoneDB	LR local constraint
SpatialDM	CCC	ST	Permutation test	Uses cell-type deconvolution as post-analysis	CellChatDB	LR constraint: spatial weight $\exp (-d^{2})$
MISTy	CCC/CCI	ST	Random forest	Uses dominant cell type	–	LR constraint: multi-scale spatial weight
SVCA	CCC/CCI	ST	Variance components model	–	–	LR constraint: spatial weight $\exp (-d^{2})$
NicheDE/LR	CCC/CCI	ST	Negative binomial regression	Uses deconvolved cell-type proportion as input	NicheNetDB	LR constraint: spatial weight $\exp (-d^{2})$; cell-type enrichment in spatially local niche
Spacia	CCC/CCI	ST	Bayesian logit regression	–	–	LR constraint: Bayesian learnable spatial weight
COMMOT	CCC	ST	Collective OT + permutation	Uses dominant cell type	CellChatDB, scSeqComm	LR constraint: spatial weight $\exp (-d^{2})$ + hard threshold
SPIDER	CCC	ST	OT + SVI test	Uses dominant cell type	CellTalkDB, KEGG, Reactome	LR constraint: spatial weight $\exp (-d^{2})$
CLRIA	CCC	ST	Unbalanced OT + permutation	Brain region annotation, not cell type	Not specified	LR constraint: neuron connected long-distance CCC, based on fMRI
SCOTIA	CCC	ST	Unbalanced OT + permutation	–	CellPhoneDB, CellChatDB	LR constraint: spatial weight $\exp (-d^{2})$
HoloNet	CCC	ST	GNN	Uses cell-type deconvolution as input	CellChatDB, ConnectomeDB, SignaLink, TRRUST	LR constraint: spatial weight $\exp (-d^{2})$
NicheCompass	CCC/CCI	ST	GVAE	Uses dominant cell type	OmniPath, MEBOCOST, CollecTRI, NicheNetDB	LR constraint: local KNN; microenvironment-specific CCC
CellNEST	CCC	ST	GAT + contrastive learning	Uses dominant cell type	CellChatDB, NicheNetDB	LR constraint: 2-hop; microenvironment-specific CCC
SpaTalk	CCC	ST	Knowledge graph	Reconstructs single-cell resolution on spot	CellTalkDB, KEGG, Reactome, AnimalTFDB	–
FlowSig	CCC	scRNA/ST	Completed partial DAG	Uses dominant cell type	OmniPath	–
Tensor-cell2cell	CCC	scRNA/ST	Tensor decomposition	Uses dominant cell type	CellChat	–
TraSig	CCC	scRNA	Permutation test	–	FANTOM5	–
TimeTalk	CCC	scRNA	Permutation test	–	CellTalkDB	–
NicheNet	CCC	scRNA	Graph propagation	–	In-house NicheNetDB	–
MultiNicheNet	CCC	scRNA	Multi-task weighted graph propagation	–	In-house NicheNetDB	–
scMLnet	CCC	scRNA	Network analysis	–	In-house scMLnetDB	–
stMLnet	CCC	ST	Network analysis	Uses dominant cell type	In-house stMLnetDB	LR constraint: spatial weight *1/d*; infers downstream TF and TG activity
CCCvelo	CCC	ST	Multilayer network + ODE + DNN	–	stMLnetDB	LR constraint: spatial weight *1/d* and Delaunay triangulation neighbor; infers downstream TF and TG activity

### Statistical inference under spatial constraints

In ST data, many approaches employ statistical inference to quantify the likelihood that ligands expressed by sender cells or cell types are responsible for the observed molecular states of cognate receptors in receiver cells or cell types within a plausible spatial range. Methods such as CellChat [2], stLearn [32], and SpatialDM [33] explicitly model LR signaling strength and use permutation-based tests to assess whether LR co-expression is significantly associated with cell type or spatial location, thereby deriving empirical *p*-values. Spacia [34] integrates spatial location, cell-type information, and broad signaling gene expression into a unified Bayesian framework to identify interacting individual cells between two cell types within spatial proximity, yielding Bayesian posterior predictive *p*-values. In addition, SVCA [35], MISTy [36], and NicheDE [37] apply frequentist regression-based models to decompose variation in signaling gene expression into intrinsic and extrinsic effects, where the extrinsic component provides the basis for CCC analysis by capturing associations with neighboring cell types or spatial context. Overall, statistical inference-based frameworks offer high interpretability and are grounded in rigorous statistical testing, making them well suited for hypothesis-driven CCC analysis.

#### Cell–cell communication inference with permutation-based hypothesis tests

In the absence of ground truth for CCC activity, many methods adopt permutation-based hypothesis testing to identify LR interaction patterns that deviate from random configurations. The core assumption underlying these approaches is that the permutation procedure generates a null distribution corresponding to a scenario with no LR interaction. Different methods implement this idea in distinct ways by permuting different aspects of the data. For example, CellChat-v2 [2] permutes cell-type labels to disrupt the association between gene expression and cell identity, SpatialDM [33] permutes spatial locations to break gene-location dependence, and stLearn [32] combines both strategies, using non-interacting genes to construct location-based null distributions and permuting cell-type labels for cell-type-specific tests.

Early CCC methods developed for scRNA-seq data, such as CellChat-v1 [2] and CellPhoneDB [38], infer communication by aggregating ligand and receptor expression at the cell-type level. While effective in non-spatial settings, this strategy ignores the biophysical constraint that paracrine and juxtacrine signaling is limited to nearby cells. CellChat-v2 partially addresses this limitation by incorporating spatial information through a cell-type-level distance, defined as the truncated mean of nearest-neighbor distances between cells of two types. LR signaling strength is then computed as the product of mean ligand and receptor expression weighted by a distance-decay function. Although this reduces false positives between distant cell-type pairs, aggregation at the cell-type level can still mix signals from spatially distant cells that are unlikely to communicate.

More recent methods impose spatial constraints at the single-cell level prior to aggregation. For instance, stLearn [32] defines local spatial neighborhoods and quantifies LR signaling strength based on the extent of spatial co-localization between ligand-expressing sender cells and receptor-expressing receiver cells. This formulation more closely reflects short-range communication and reduces noise from distant cells. However, it primarily captures enrichment of LR co-localization rather than expression magnitude, treating weakly and strongly expressed but co-localized LR pairs equivalently.

SpatialDM [33] further integrates expression magnitude by weighting spatially co-localized LR pairs according to expression coherence. Specifically, it defines LR signaling strength using bivariate Moran’s *I* [39] combined with an exponential distance-decay function, yielding a metric that jointly captures spatial proximity and co-expression.

Despite these methodological differences, permutation-based CCC methods can be broadly divided into two classes. Cell-type- or cluster-specific permutation tests assume that LR expression is independent of cell-type labels under the null and are, therefore, sensitive to interactions enriched in specific sender–receiver cell-type pairs, but may overlook region-specific interactions involving mixed or fine-grained subpopulations [32, 40]. In contrast, LR co-localization-based permutation tests assume spatial independence between ligand and receptor expression and are more sensitive to spatially heterogeneous interaction patterns localized to specific tissue regions [33, 40]. Notably, a spatially co-localized and co-expressed LR pair is not necessarily cell-type specific, highlighting the distinct signals captured by these permutation strategies.

#### Cell–cell communication inference with Bayesian inference and machine learning

Rather than relying on permutation tests, some methods explicitly model how signaling gene expression relates to spatial variations in neighbor cell distribution, cell-type composition, and other signaling genes and then quantify the strength of these associations through regression parameters. The central assumption underlying these methods is that gene expression can be decomposed into cell-state- or cell-type-specific intrinsic components and intercellular extrinsic components. For example, the chemokine *CXCL12* is the cognate ligand of the receptor *CXCR4*, whereas the *CXCL12-*γ ** isoform has been observed in the nuclear region, suggesting that chemokines may also exert cell-intrinsic functions that are unrelated to CCC [41]. Regression-based frameworks are therefore typically designed for broad CCI analysis, as they model the spatial variation of all genes, including both signaling genes involved in CCC and genes that do not directly participate in intercellular interactions. In this review, we focus specifically on the application of these regression-based methods to signaling genes, in order to align with the scope of CCC analysis.

MISTy [36] dissects spatial variation in cellular organization using a multiview framework. Specifically, it defines an *intraview* that captures the effects of intracellular signaling genes within the cell itself, a *juxtaview* that models the influence of signaling genes in immediately adjacent (one-hop) neighboring cells, and a *paraview* that represents the effects of signaling genes in a broader neighborhood within a predefined spatial radius. In addition, MISTy allows users to define optional custom views to incorporate other sources of information. MISTy uses random forest models to associate the expression of a target signaling gene, denoted by $Y_{\operatorname{target}}$, with view-specific features $G_{v}(\tilde{Y}, S, T)$ derived from other predictor signaling genes $\tilde{Y}$ in each view, where *S* denotes cell spatial location, and *T* denotes additional prior information; e.g. MISTy can incorporate cell-type annotations by defining cell-type-based views, enabling estimation of cell-type-level contributions to CCC within each view. The importance of each target–predictor pair is then quantified within each view and weighted by a view-level importance parameter $\alpha _{v}$, yielding the following model:


\begin{align*} & Y_{\operatorname{target}} = \alpha_{\operatorname{intercept}} + \alpha_{0} F_{0} + \alpha_{\operatorname{intra}} F_{\operatorname{intra}} + \alpha_{\operatorname{juxta}} F_{\operatorname{juxta}} + \alpha_{\operatorname{para}} F_{\operatorname{para}}, \end{align*}

where $F_{v}$ for $v \in \{0, \operatorname{intra}, \operatorname{juxta}, \operatorname{para}\}$ represents the random forest model trained on the corresponding view-specific feature $G_{v}$.

Although MISTy provides a flexible and general framework for modeling CCI, its direct application to CCC analysis has several limitations. First, MISTy is designed to capture statistical associations between target and predictor signaling genes across spatial neighborhoods (views) of varying sizes; however, the appropriate spatial hyperparameters defining these views can be difficult to estimate. Second, the detected associations reflect generic gene–gene relationships and cannot be directly interpreted as LR-mediated CCC events without explicitly incorporating prior knowledge, such as curated LR pair databases or downstream TF-target regulatory networks. Finally, because spatially adjacent cells often exhibit highly correlated juxtaview and paraview features $G_{v}$, feature redundancy can reduce effective feature variability, thereby diminishing random forest diversity and potentially compromising model generalization.

To address the statistical challenges posed by spatial dependency, SVCA [35] applies spatial variance component analysis within a linear mixed model framework to explicitly model spatial dependence using variance components. SVCA [35] decomposes variation in target signaling gene expression into intracellular and intercellular components at the level of variance, formulated as


\begin{align*} &Y_{\operatorname{target}} \sim N(0, \tau_{\operatorname{int}}K_{\operatorname{int}} + \tau_{\operatorname{spatial}}K_{\operatorname{spatial}} + \tau_{\operatorname{ccc}}K_{\operatorname{ccc}} + \sigma^2_\epsilon I)\end{align*}

Analogous to the intraview component in MISTy, the cell-similarity kernel $K_{\operatorname{int}}$ captures intracellular associations between the target signaling gene and other signaling genes within the same cell. The kernel $K_{\operatorname{spatial}}$ captures spatial autocorrelation effects that depend solely on cell location and are independent of signaling gene expression. In contrast, $K_{\operatorname{ccc}}$ captures associations between the target signaling gene and other signaling genes weighted by an exponential decay function of intercellular distance, such that signaling genes expressed in spatially proximal cells exhibit stronger associations with the target gene, whereas those in distant cells contribute less. The parameters $\tau _{\operatorname{int}}$, $\tau _{\operatorname{spatial}}$, $\tau _{\operatorname{ccc}}$, and $\sigma ^{2}_{\epsilon }$ quantify the strength of each variance component.

Owing to its statistically robust variance-component formulation, SVCA can, although not explored in the original work, be naturally extended to support formal hypothesis testing of $H_{0}:\tau _{\operatorname{ccc}}=0$, thereby assessing whether CCC-related variance contributes significantly to the expression of the target signaling gene. However, variance component models are typically computationally intensive, and scaling SVCA to high-resolution ST datasets, which often contain tens of thousands of cells [42], can be challenging. In addition, the tuning parameter governing the spatial decay of cell–cell distance, intended to capture juxtacrine and paracrine communication constraints, remains difficult to estimate. Moreover, SVCA does not support the integration of additional prior information, such as cell-type annotations, which can limit its interpretability in practical biological applications.

In contrast, NicheDE [37] explicitly employs a negative binomial regression model to decompose gene expression into cell-type-specific intrinsic effects and local neighborhood (niche) cell-type enrichment effects, with an emphasis on cell-type-related communication. In addition, NicheDE incorporates a CCC-focused module, NicheLR, which restricts the analysis to ligands and leverages ligand–target regulatory networks from NicheNet [43] to prioritize ligands that contribute to functionally relevant communication (see NicheNet in Section Linking cell–cell communication to downstream functional responses). Suppose there are *N* cells in an ST dataset with *M* cell types. NicheLR assumes that the ligand expression $Y_{\operatorname{ligand},c}$ of a sender cell *c* depends not only on the sender cell type $T_{c}$ but also on the enrichment of neighboring receiver cell types, denoted by $N_{\sigma ,c,T^{(k)}}$ for $k \in \{1,\ldots ,M\}$. This neighborhood cell-type enrichment is defined as the abundance of each cell type $T^{(k)}$ in the spatial neighborhood of the index cell *c*, weighted by an exponential decay function of intercellular distance. Accordingly, NicheLR defines the spatial “niche” of cell *c* as an *M*-dimensional vector that quantifies the enrichment of all surrounding cell types. NicheLR formulates ligand gene expression using a negative binomial regression model with dispersion parameter $\gamma _{\operatorname{ligand}}$, such that


\begin{align*} &\log \operatorname{E}\!\left(Y_{\operatorname{ligand},c}\mid T_c, N_{\sigma,c}\right) = \log \mu_{\operatorname{ligand},T_c} + \sum_{k=1}^M N_{\sigma,c,T^{(k)}}\, \beta_{\sigma,T_c,T^{(k)}}^{\operatorname{ligand}}, \end{align*}

where $\mu _{\operatorname{ligand},T_{c}}$ captures sender cell-type-specific intrinsic variation and $\beta _{\sigma ,T_{c},T^{(k)}}^{\operatorname{ligand}}$ quantifies the contribution of niche cell-type enrichment.

Despite the complexity of the notation, the underlying idea of NicheLR can be illustrated with a simple example. In hematopoietic stem cell (HSC) quiescent tissue regions, HSCs are expected to be spatially enriched near bone marrow stromal cells that highly express *CXCL12*, and less enriched near stromal cells with low *CXCL12* expression, given that the *CXCL12*–*CXCR4* LR pair is essential for maintaining HSC quiescence [44]. In heterogeneous tissues, stromal cells that are not co-localized with HSCs primarily contribute to estimating the intrinsic effect $\mu _{\operatorname{ligand},T_{c}}$, whereas stromal cells that are co-localized with HSCs and exhibit high *CXCL12* expression inform the estimation of the cell-type enrichment effect $\beta _{\sigma ,T_{c},T^{(k)}}^{\operatorname{ligand}}$. NicheLR derives statistical significance for cell-type enrichment effects by testing the null hypothesis ${H_{0}}: \beta _{\sigma ,T_{c},T^{(k)}}^{\operatorname{ligand}} = 0$ for $k \in \{1,\ldots ,M\}$. Failure to reject this hypothesis for all cell types indicates the absence of cell-type-informative intercellular communication. In contrast, when specific cell types significantly contribute to ligand expression variation, NicheLR further applies NicheNet to infer potential functional communication through ligand–target regulatory networks. However, because the association between ligand expression and niche cell-type enrichment is estimated globally, it remains unclear which individual cells or spatial subregions drive the inferred communication, particularly in the absence of explicit receptor co-expression modeling. Moreover, optimizing the spatial decay hyperparameter *σ * that governs niche definition remains challenging.

Spacia [34] jointly addresses the challenges of spatial neighborhood size selection and cell-type-specific communication using a unified Bayesian framework based on multiple-instance learning. Although Spacia was originally developed as a CCI inference method that considers all genes, here we focus on its “no aggregation” or “knowledge-driven aggregation” modes, in which only signaling genes from curated LR or pathway databases are included. To identify sender cells that genuinely interact with a receiver cell, Spacia introduces the concept of *primary senders*, defined as cells that are not only spatially co-localized with the receiver cell but also exhibit high ligand co-expression. These are distinguished from *non-primary senders*, which are unlikely to communicate with the receiver cell even if they belong to the same cell type. Rather than defining spatial neighborhoods using a predefined fixed radius, as in stLearn [32] and CellChat-v2 [2], or a continuous distance-based decay function with user-defined bandwidth, as in SpatialDM [33] and NicheLR [37], Spacia adaptively selects primary sender cells through a learnable indicator function modeled via Bayesian probit regression. This design alleviates the need for manual hyperparameter tuning. Subsequently, Spacia fits a second Bayesian probit regression model to estimate the effects of one or multiple ligands expressed in primary sender cells on the receiver cell, which is interpreted as communication strength, and derives Bayesian posterior *p*-values to support statistical inference. For each cell-type pair and the corresponding cell-type-specific LR pair, Spacia iteratively updates the two nested Bayesian probit regression models with MCMC algorithms, jointly estimating communication strength and primary sender assignment. As a result, compared with other methods, Spacia not only infers global cell-type-specific communication strength but also explicitly identifies which individual cells within a given cell-type participate in intercellular communication. This capability advances CCC analysis from cell-type-level conclusions to intra-cell-type resolution, enabling more detailed characterization of communication mechanisms across distinct cell states or microenvironments.

### Optimal transport framework for many-to-many cell–cell communication modeling

The aforementioned statistical inference methods typically model intercellular communication independently for each cognate LR pair. However, individual cells can simultaneously interact with multiple neighboring cells through diverse LR species [11]. This inherently many-to-many structure requires a modeling framework that captures joint coupling between sender and receiver cell populations rather than enforcing pairwise assignments. OT [45] provides a mathematically principled and flexible framework for estimating many-to-many communication patterns through a unified optimization problem that maps probability distributions from senders (ligands) to receivers (receptors), while naturally integrating spatial and biological constraints.

#### Mathematical formulation of optimal transport

Consider a simplified setting with a single LR pair, *N* sender cells, and *M* receiver cells. Let the observed ligand expression in sender cells be denoted by $\mathbf{L} \in \mathbb{R}_{+}^{N}$, and the observed receptor expression in receiver cells by $\mathbf{R} \in \mathbb{R}_{+}^{M}$. The OT problem can be formulated as


\begin{align*} & \mathbf{P} = \arg\min_{\mathbf{P} \in \Gamma} \langle \mathbf{P}, \mathbf{C} \rangle_{F} - \epsilon H(\mathbf{P}), \end{align*}

where $\langle \mathbf{P}, \mathbf{C} \rangle _{F}$ denotes the Frobenius inner product between the transport cost matrix $\mathbf{C} \in \mathbb{R}_{+}^{N \times M}$ and the transport plan $\mathbf{P} \in \mathbb{R}_{+}^{N \times M}$. Here, $H(\mathbf{P})$ is an entropy regularizer, and *ε> 0* controls the regularization strength; this entropic regularization renders the optimization problem strongly convex and computationally tractable. The entry $[\mathbf{C}]_{i,j}$ represents the cost of communication between sender cell *i* and receiver cell *j*, with higher cost assigned to spatially distant cell pairs and lower cost to nearby pairs. Correspondingly, $[\mathbf{P}]_{i,j}$ represents the strength of the latent signaling flow from sender cell *i* to receiver cell *j*. To ensure consistency between the inferred signaling flow and the observed ligand and receptor expression, the transport plan $\mathbf{P}$ is constrained to lie in the feasible set *Γ *, which specifies how the marginal distributions of $\mathbf{P}$ relate to $\mathbf{L}$ and $\mathbf{R}$.

Intuitively, OT-based methods seek an efficient transport plan $\mathbf{P}$ to transport ligand signals to receptors, under a predefined cost matrix $\mathbf{C}$. The cost matrix is typically designed to reflect biological assumptions, such as assigning higher costs to long-distance communication to penalize unrealistic ligand diffusion. Additional mathematical constraints can also be incorporated into *Γ * to ensure the optimization problem is computationally tractable.

#### Collective optimal transport incorporates cell competition

A single receptor can bind multiple ligands secreted by neighboring sender cells; e.g. *EGFR* has seven known ligands [46]. To capture this many-to-one signaling mechanism, COMMOT [47] introduces a collective optimal transport (COT) framework that jointly optimizes signaling flow across all LR pairs and cells. Extending the notation of the toy example, let there be $n_{l}$ ligand species and $n_{r}$ receptor species. Accordingly, the transport plan $\mathbf{P}$ and cost tensor $\mathbf{C}$ are 4D arrays in $\mathbb{R}_{+}^{n_{l} \times n_{r} \times N \times M}$. COMMOT incorporates spatial information into the transport cost tensor $\mathbf{C}$ to enforce local communication constraints, defined as


\begin{align*} & [\mathbf{C}]_{i,j} = \begin{cases} \phi(d_{i,j}), & d_{i,j} \le T_{l,r}, \\ \infty, & \textrm{otherwise}, \end{cases} \end{align*}

where $d_{i,j}$ denotes the spatial distance between sender cell *i* and receiver cell *j*, and $\phi (\cdot )$ is a squared or exponential cost function. Cell pairs separated by distances exceeding the LR-specific spatial limit $T_{l,r}$ are assigned infinite cost, reflecting the assumption that distant cells are unlikely to communicate. Although $T_{l,r}$ may in principle vary across LR species, COMMOT uses a uniform and relatively large spatial limit in practice, as accurate species-specific estimation remains challenging.

To model cell competition, COMMOT imposes hard marginal constraints on the transport plan,


\begin{align*} & \Gamma_{\textrm{commot}} = \Bigl\{ \mathbf{P}: \mathbf{P}_{l,r,\cdot,\cdot} = \mathbf{0} \;\textrm{for}\; (l,r) \notin I,\; \sum_{r,j} \mathbf{P}_{l,r,i,j} \le L_{l,i},\; \sum_{l,i} \mathbf{P}_{l,r,i,j} \le R_{r,j} \Bigr\}, \end{align*}

where *I* denotes the set of cognate LR pairs. If ligand *l* cannot bind receptor *r*, i.e. *(l,r)∉ I*, the corresponding signaling flow $\mathbf{P}_{l,r,\cdot ,\cdot }$ is constrained to zero. Intuitively, this marginal constraint ensures that the total signaling flow sent from sender cell *i* through ligand *l* to all receiver cells does not exceed the observed expression level of ligand *l* in cell *i*. Similarly, the total signaling flow received by receiver cell *j* through receptor *r* from all nearby sender cells should not exceed the observed expression level of receptor *r* in cell *j*.

In this way, COMMOT explicitly models competition among ligands and receptors for binding. Under this formulation, the transport plan $\mathbf{P}$ represents latent signaling strengths, with the cost tensor encoding spatial proximity and the marginal constraints encoding biochemical competition. COMMOT further derives cell-level vector fields from $\mathbf{P}$ to visualize interpolated communication directions for individual LR pairs across tissue space. Unfortunately, the transport plan alone does not directly identify which LR interactions are cell-type- or cluster-specific, nor which are spatially structured. To address this, COMMOT applies permutation-based testing by disrupting associations between aggregated cluster-level signaling strength and cell-type labels, as well as by permuting spatial locations within or across clusters. Consequently, although COMMOT explicitly captures many-to-many competition among cells, statistical inference remains dependent on permutation strategies and is therefore subject to the associated uncertainties in heterogeneous tissues.

SPIDER [40] extends the COT framework by introducing *interaction-interface*-based constraints that restrict intercellular communication according to each cell’s interaction capacity, defined as the total expression of ligands and receptors. In this formulation, cells with higher interaction capacity are assigned larger spatial interfaces, allowing them to potentially communicate with more neighbors. As a result, SPIDER replaces the distance-decay cost used in COMMOT with an indicator cost function defined over interaction interfaces. Unlike COMMOT, where neighborhood size is controlled by LR-specific spatial limits $T_{l,r}$, SPIDER defines interaction interfaces at the cellular level, which are shared across all LR species. After estimating the transport plan under interface constraints, SPIDER aggregates latent signaling strength into *abstract interfaces*, each representing a group of spatially adjacent interfaces. To identify spatially heterogeneous LR interactions, SPIDER adapts the concept of spatially variable genes (SVGs) and extends tests such as SPARK-X [48] and SpatialDE [49] to spatially variable interaction (SVI) analysis, replacing gene expression with abstract-interface-level LR signaling strength. Unlike location permutation tests, SVI analysis directly assesses spatial autocorrelation in LR interaction patterns, thereby avoiding additional uncertainty introduced by permutation. SPIDER further correlates SVIs with receptor-regulated TFs to identify interactions supported by downstream functional signaling.

#### Unbalanced optimal transport relaxes competition constraint

COT-based methods incorporate cell competition by imposing restrictive marginal constraints such as $\Gamma _{\textrm{commot}}$, which require the total latent signaling strength to be no greater than the observed expression. However, mRNA expression measured from ST data does not faithfully reflect the actual protein abundance of ligands and receptors and is further affected by technical noise such as dropout. To address this discrepancy, CLRIA [50] and SCOTIA [51] adopt an unbalanced OT (UOT) framework. Extending the notation from the toy example, UOT is formulated as


\begin{align*}& \mathbf{P}= \mathop{\arg\min}_{\mathbf{P} \in \mathbb{R}_{+}} \langle \mathbf{P}, \mathbf{C} \rangle_{F} - \epsilon H(\mathbf{P}) + \tau_{a} \textit{KL}(\mathbf{P}\mathbf{1}_{M} \mid \mathbf{L}) + \tau_{b} \textit{KL}(\mathbf{P}^{\top}\mathbf{1}_{N} \mid \mathbf{R}), \end{align*}

where the Kullback–Leibler (KL) divergence terms act as soft constraints that quantify the mismatch between the marginal distributions of latent signaling strength and the observed ligand and receptor expression. Intuitively, the discrepancy from $\Gamma _{\textrm{commot}}$ arises because the KL regularization terms relax the hard expression constraints used in $\Gamma _{\textrm{commot}}$. Since observed ligand and receptor expression may be affected by technical noise and dropout, the inferred signaling activity is allowed to slightly exceed the observed expression levels, rather than being strictly bounded by the observed ligand or receptor expression as in $\Gamma _{\textrm{commot}}$.

The aforementioned methods are generally grounded in the assumption that CCC occurs primarily through local mechanisms such as autocrine, juxtacrine, and paracrine signaling. In the brain connectome, however, the presence of long-range fiber tracts enables both proximal and distal communication between anatomically distant regions, shifting the focus from strictly co-localized communication to interregional signaling [52]. To capture this property, CLRIA [50] leverages the Brainnetome atlas [53] to define brain regions and constructs a brain network communication model based on diffusion MRI-derived structural connectivity. The transport cost matrix is defined using the mean first passage time of a biased random walk, enabling the model to capture both short- and long-range signaling efficiency. Another key contribution of CLRIA is its low-rank factorization of the 3D transport plan,


\begin{align*} & \mathbf{P} = A \operatorname{diag}(C^\top \mathbf{L}_{|I|}) B^\top \in \mathbb{R}_{+}^{N \times N \times |I|}, \end{align*}

where *|I|* denotes the number of cognate LR species, and *N* is the number of cells. This factorization substantially reduces computational complexity and encourages LR pairs to share low-dimensional communication patterns, which is consistent with the biological observation that many signaling pathways rely on common brain-wide communication motifs. Downstream analyses in CLRIA focus on identifying asymmetric signaling between brain regions by testing whether LR communication from region *i* to region *j* is significantly stronger than the reverse direction.

In contrast to jointly constructing OT transport plans across multiple LR species, SCOTIA [51] constructs separate UOT models for individual LR pairs between specific sender and receiver cell types, with the goal of identifying differentially enriched cell-type-specific LR interactions between treated and untreated conditions. Rather than constructing $\mathbf{P}$ using all $N_{s}$ sender and $N_{r}$ receiver cells, SCOTIA applies DBSCAN [54] to cluster each cell type into spatial subclusters and retains only spatially adjacent sender and receiver subclusters for downstream analysis. For the remaining $n_{s}$ sender cells and $n_{r}$ receiver cells, SCOTIA constructs a UOT model for each LR pair and assigns transport costs as $[\mathbf{C}]_{i,j} = d_{i,j} / \sqrt{L_{i} R_{j}}$. After estimating the transport plan $\mathbf{P} \in \mathbb{R}_{+}^{n_{s} \times n_{r}}$, SCOTIA first applies permutation testing to identify robust LR pairs by disrupting associations with gene expression and spatial location. Differentially enriched LR interactions are then identified using Mann–Whitney U-tests or mixed-effects models, with sample identity treated as a random effect and treatment status as a fixed effect.

Although SCOTIA enables condition-specific CCC analysis, its permutation strategy, which is restricted to spatially adjacent subclusters, may raise statistical concerns. In particular, null distributions constructed from only $n_{s} + n_{r}$ subcluster cells can differ substantially from those generated using larger and more diverse cell pools, as adopted in other methods. Intuitively, larger cell pools better capture expression heterogeneity and yield more stable null distributions of non-interacting cells. Restricting permutations to limited spatial neighborhoods may therefore underestimate background variability and compromise statistical power.

Overall, compared with statistical inference approaches, OT-based methods are well suited for modeling many-to-many CCC through hard or soft marginal constraints. With appropriately designed transport costs that account for short- or long-range communication, OT-based frameworks yield cell-level transport plans that reduce contributions from biophysically implausible interactions. However, OT alone does not inherently identify cell-type-specific or spatially variable LR interactions. Consequently, OT-based CCC methods typically require an additional statistical layer, such as permutation-based testing or regression-based SVI analysis, to support context-specific inference.

### Learning spatial cell–cell communication representations with deep learning

Although statistical inference- and OT-based approaches have shown strong capability in identifying intercellular LR interactions, deep learning-based methods contribute to expanding the scope of CCC downstream analysis. HoloNet [55], a GNN-based framework, offers a natural advantage for integrating intracellular gene–gene regulatory structures into CCC analysis. CellNEST [56], which combines graph attention networks (GATs) with the contrastive learning framework deep graph infomax (DGI), introduces the concept of spatially multi-hop communication. NicheCompass [57], based on a multimodal conditional graph variational autoencoder (GVAE), identifies spatial niches defined by CCC signaling gene programs rather than by cell-type composition.

#### Incorporating intracellular signaling information

HoloNet [55] aims to disentangle functional CCC events by determining which LR pairs can effectively modulate target gene expression in receiver cells among all spatially interacting LR species. This is achieved using a multi-view GNN architecture, in which each LR species is treated as a separate view and an attention mechanism quantifies its contribution to target gene prediction. For each view, HoloNet constructs a cell-level graph whose edges are weighted by LR co-expression scores modulated by an exponential spatial decay function of intercellular distance. HoloNet further decomposes target gene expression into cell-intrinsic and LR-associated components, assuming that only the LR-associated component is regulated by intercellular communication. Model parameters are optimized by minimizing the mean squared error between predicted and observed target gene expression. Despite the advantage of incorporating target gene regulation beyond solely LR interaction, HoloNet still has several limitations. First, LR–TG regulation oversimplifies signaling cascades, TF activation, and broader gene regulatory networks. Second, the LR-pair-specific multi-view design substantially increases model complexity, making HoloNet potentially prone to overfitting when applied to spot-level ST datasets with limited spot numbers. Third, HoloNet models each target gene independently, which may limit its ability to capture gene-gene dependencies or pathway-level regulatory effects induced by CCC.

#### Spatially multi-hop communication

Beyond single-layer LR interactions, CellNEST [56] introduces cell-level multi-hop relay-network communication detection to identify putative L-R-L-R signaling patterns. Because no ground truth exists for relay networks in ST data, CellNEST trains a GAT [58] encoder using DGI [59]-based contrastive learning. CellNEST first constructs a spatial neighborhood graph in which two cells are connected if they lie within a fixed spatial radius and exhibit elevated ligand and receptor expression. Each LR pair corresponds to a distinct edge, allowing multiple edges between the same pair of cells, with edge features including intercellular distance, LR co-expression scores, and LR identity. Contrastive learning is then applied by comparing the original spatial graph with a corrupted version in which spatial relationships are randomly permuted. Through this process, the GAT assigns high attention scores to edges representing strong and consistent neighborhood relations, and CellNEST retains the top 20% highest-attention edges as high-confidence CCC events. From these edges, CellNEST quantifies the frequency of two-hop L-R-L-R relay networks and extends the analysis to multi-hop relays. For example, CellNEST identified an abundant relay from the T cell homing signal *CCL19-CCR7* to the T cell trafficking signal *CCL21–CXCR4* in T cell zones, suggesting a potential multi-step mechanism underlying T cell migration. Compared with single LR co-expression, multi-hop relay patterns are less likely to reflect false-positive CCC events.

Despite this innovation, multi-hop CCC inference remains methodologically challenging. Although CellNEST introduces multi-hop relay-network communication and can in principle be extended beyond two-hop paths, its practical analysis mainly focuses on two-hop L-R-L-R relays. As the relay depth increases, the search space expands rapidly, and weakly supported or false-positive LR edges may be propagated and amplified across downstream steps. This creates a key bottleneck in balancing the depth of inferred communication chains with the statistical robustness of each interaction. Moreover, CellNEST relies on a DGI-based contrastive learning framework in which the original spatial graph is compared with a globally corrupted graph. While this design is useful in the absence of ground-truth relay networks, the biological meaning of the corrupted graph remains ambiguous, especially in heterogeneous tissues. Although CellNEST further uses PPIs, ligand–target relationships, and TF–target gene interactions for *post hoc* confidence scoring, these priors are not directly incorporated into GAT/DGI-based edge learning or relay path discovery. Therefore, it remains difficult to mechanistically explain why specific relay paths receive high attention scores, and this ambiguity may become more severe in longer multi-hop analyses.

From a biological perspective, the L-R-L-R formulation is still a simplified abstraction of cascading signaling. In realistic tissues, a ligand from Cell A may activate a receptor on Cell B, trigger intracellular signaling and transcriptional programs, induce downstream target genes including a secondary ligand, and subsequently signal to Cell C. Thus, future multi-hop CCC models may benefit from multilayer network analysis (Section Spatially induced multilayer signaling analysis) that jointly incorporates LR interactions, receptor-to-TF signaling, TF-to-target regulation, and ligand-induced response programs. Such constraints could reduce noise amplification and improve biological interpretability. Another promising direction is to integrate CCC priors with recurring multicellular spatial pattern detection. For example, tertiary lymphoid structures are often composed of coordinated T cell, B cell, and dendritic cell compartments, and such multicellular spatial units are closely related to immune communication and cell recruitment. TrimNN [60] identifies multicellular spatial units based solely on cell-type and location information. Combining this type of spatial-unit detection with CCC prior knowledge may better control noise amplification while capturing organized multicellular relay programs.

#### Cell–cell communication-driven spatial niches

Beyond identifying individual LR interactions or multi-hop relay networks, NicheCompass [57] shifts the focus to learning cell embeddings and spatial niches that reflect communication-defined microenvironments, rather than clusters defined solely by cell type or tissue structure. For example, NicheCompass subdivides broad annotations such as *Forebrain/midbrain/hindbrain* into higher-resolution niches, including *Gdf10/Fgf3+ hindbrain*, *Calca/Shh+ floor plate*, *Fgf17/Efna2+ midbrain*, and *Pcsk1n/Dkk1+ forebrain*, each characterized by interpretable signaling gene programs. The method constructs a spatial neighborhood graph using either *k*-nearest neighbors or fixed-radius proximity to approximate juxtacrine and paracrine interactions, and incorporates domain knowledge from intercellular and intracellular signaling pathways to constrain latent embeddings. Each embedding dimension is encouraged to represent the activity of a specific signaling program, while allowing the model to learn *de novo* spatial programs absent from prior knowledge. To model intercellular communication, each signaling program is decomposed into a self-component and a neighborhood component. Prior knowledge of intracellular signaling, including receptors, TFs, and downstream target genes, is encoded in the self-component, whereas ligands and other sender-side genes involved in outgoing signaling are assigned to the neighborhood component. A conditional variational graph autoencoder with attention-based message passing learns which neighboring cells are most likely to interact with each focal cell, and clustering the resulting embeddings yields communication-defined niches. NicheCompass emphasizes communication-defined cellular niches by embedding cells according to coordinated signaling programs. This niche-centric perspective enables quantitative characterization of spatial microenvironments shaped by intercellular communication, rather than resolving discrete LR interactions or relay paths alone. In contrast to spatial clustering methods such as BANKSY [61] and spatiotemporal niche trajectory methods such as NicheFlow [62] that rely on general gene expression information, NicheCompass explicitly incorporates CCC related prior knowledge and identifies niches based on shared sender–receiver signaling programs that may span multiple cell types. Because each latent dimension corresponds to a known or *de novo* signaling program, niche identities are biologically interpretable, and the separation of self and neighborhood components inherently encodes communication directionality, supporting downstream analyses of niche–niche interactions. However, NicheCompass has some limitations. In ST platforms with limited gene panels, incomplete coverage of ligands, receptors, and downstream genes may weaken the interpretability and shift niche annotation toward *de novo* programs or dominant cell-type composition. Moreover, NicheCompass may be sensitive to hyperparameter choices, particularly the seven regularization and loss weighting terms in its loss function. In practice, the number of *de novo* gene programs introduces a trade-off between capturing dataset-specific patterns and maintaining biological interpretability. Therefore, the performance and interpretability of NicheCompass may depend on hyperparameter settings in real-world applications.

In summary, while these deep learning frameworks transition CCC analysis from simple spatial co-expression to complex and diverse downstream analysis, they face challenges in practical application. HoloNet and CellNEST represent purely data-driven architectures that offer high flexibility but suffer from low interpretability and a susceptibility to overfitting on technical noise. Conversely, NicheCompass mitigates the interpretability issue by integrating prior knowledge, yet its complex GVAE structure amplifies sensitivity to hyperparameter tuning and data sparsity. Across all three methods, the lack of large-scale, unified ST benchmarks hinders robust cross-dataset generalization, often rendering inferred downstream regulatory signals or niche boundaries unstable when processing subcellular-level datasets with a small gene panel.

### Spatially induced multilayer signaling analysis

While the majority of CCC inference methods primarily rely on transcript-level LR co-expression as the primary evidence of communication, real cellular signaling involves multiple regulatory layers beyond LR transcription. These include protein/peptide-level ligand secretion, receptor activation, intracellular post-translational signaling mechanisms such as kinase cascades and TF activation [63], chromatin-level regulatory processes such as chromatin accessibility remodeling [64], and downstream target gene(TG) regulation. To mitigate the bias between RNA expression level LR coexpression and true cellular signaling, some studies have attempted to model signal transmission more accurately by explicitly integrating these multilayer signaling analyses.

Many scRNA-seq-based CCC inference methods incorporate intracellular multilayer signaling through network-based analyses. NicheNet [43], scMLnet [65], and iTALK [66] explicitly model the ligand *→ * receptor *→ * transcriptional factor *→ * target gene multilayer network, abbreviated as L-R-TF-TG network. Recently, stMLnet [67] and CCCvelo [68] extend the multilayer signaling analysis into ST data and utilize spatial location information to improve LR inference accuracy. Moreover, SpaceTravLR and CCCvelo adopt novel computational frameworks beyond network analysis.

stMLnet [67] is a spatially informed multilayer network-based CCC inference method. Compared with scMLnet, stMLnet extends multilayer CCC inference from dissociated scRNA-seq data to ST by integrating spatial coordinates with prior signaling and regulatory networks. It also organizes a comprehensive in-house CCC knowledge base, stMLnetDB, which consists of LigRecDB for LR pairs, RecTFDB for receptor–TF regulation, and TFTGDB for TF–target gene regulation. stMLnet uses spatial information to quantify distance-dependent LR signaling activity. Specifically, it models ligand diffusion rate with the steady-state and radial symmetry assumptions of ordinary differential equations (ODE), and derives a reciprocal relationship between effective ligand signal and sender–receiver distance. Furthermore, stMLnet uses random forest regression to model L-R-TG regulation in receiver cells. For each TG, spatially dependent L-R signaling activities are used as predictors of TG expression, and feature importance scores are calculated to prioritize upstream LR pairs that contribute to TG regulation. The inferred regulatory chains can be visualized using waterfall plots, which display the regulatory paths and strengths from upstream LR pairs to TFs and downstream TGs. In addition, stMLnet expands CCC downstream analysis by identifying cell-type-specific signaling feedback loops. Based on the prior L-R-TF-TG gene regulatory knowledge network, stMLnet constructs context-specific multilayer subnetworks based on the given ST data. Fisher’s exact test can be optionally used to filter inactive TFs, receptors, and their associated interactions. Given the inferred subnetworks, stMLnet searches for signaling circuits. For example, cell-type A may send ligand L1 to activate receptor R1 in cell-type B, inducing TF1-mediated expression of ligand L2; in turn, cell-type B may send L2 back to receptor R2 in cell-type A, inducing TF2-mediated expression of L1. This forms a feedback loop: Cell-type A[L1–R1–TF1–L2] *↔ * Cell-type B[L2–R2–TF2–L1]. However, unlike CellNEST, which performs cellular-level spatial multi-hop communication analysis, stMLnet identifies signaling feedback loops mainly at the cell-type level. Therefore, the feedback loop itself cannot be directly projected or visualized on the spatial tissue as a single cell-level spatial signal.

CCCvelo [68] extends multilayer signaling analysis beyond static network analysis. It formulates cell-state transition dynamics with ODEs that model latent TF activity dynamics and TG expression dynamics, and develops the deep neural network (DNN)-based PINN-CELL algorithm to estimate model parameters and latent cell-state transition time. CCCvelo combines CCC analysis with the concepts of pseudotime inference and RNA velocity. Pseudotime inference estimates a 1D latent ordering of cells, where cells at the beginning of the trajectory often correspond to stem-like or progenitor states and cells toward the end correspond to more mature or differentiated states [69, 70]. RNA velocity is a computational technique for predicting the direction and speed of cell-state transitions over time [71]. Unlike traditional pseudotime inference and RNA velocity estimation methods, CCCvelo not only predicts latent transition time and velocity directions, but also provides mechanistic interpretability of cell-state transitions through an L-R-TF-TG regulatory network. CCCvelo adopts diffusion-type LR signaling strength from stMLnet [67], using spatial decay. In addition, it incorporates contact-type LR signaling strength by using Delaunay triangulation to constrain communication to local spatial neighbors. Based on inferred LR signaling strength and the observed expression of TFs and TGs covered by stMLnetDB, CCCvelo incorporates Hill functions $\mathcal{H}(\cdot )$ into ODEs to characterize nonlinear LR–TF and TF–TG regulatory relationships:


\begin{align*} \frac{d \text{(inferred TF activity)}}{dt} &= \mathcal{H}_{\theta_{1}}( \text{all upstream LR signaling strength,}\\& \quad\textrm{ observed TF expression}, \textrm{inferred TF}\\& \quad\textrm{ activity})\\ &= \text{LR-driven TF activation} - \textrm{ TF activity}\\& \quad\textrm{decay}\\ \frac{d\text{(inferred TG expression})}{dt} &= \mathcal{H}_{\theta_{2}}( \text{inferred activity of all upstream TFs,}\\& \quad\textrm{inferred TG expression}) \\ &= \text{TF-driven TG expression change} -\textrm{ TG}\\ & \quad\textrm{expression decay}, \end{align*}

where *t* denotes latent pseudotime, *d(· )/dt* denotes the change rate of TF or TG activity, and $\theta _{1}$ and $\theta _{2}$ denote the model parameters. Specifically, the first ODE describes the dynamics of inferred TF activity, while the second ODE describes TG expression dynamics. CCCvelo then derives cell-level cell-state transition velocity from the high-dimensional TG expression velocity vector and projects it onto 2D spatial coordinates or UMAP embeddings to generate velocity arrows or streamlines, using the probabilistic mapping framework of scVelo [72]. Unlike methods such as scVelo that use spliced and unspliced RNA counts to infer velocity directions, CCCvelo derives cell velocity from L-R-TF-TG regulatory network, thereby providing mechanistic interpretability of cell-state transitions.

Although CCCvelo provides a unified framework for CCC analysis and cell-state transition inference, it also has several limitations. First, it simplifies the complexity of signaling transmission. TF activity is inferred from LR signaling and observed TF expression, and is then used to model downstream TG expression dynamics. However, biological TF–TG regulation can also be influenced by chromatin accessibility, post-transcriptional regulation and modifications, which are not explicitly modeled. Therefore, noise or bias may accumulate through the L-R-TF-TG propagation process. Second, CCCvelo relies on prior L-R-TF-TG regulatory networks, so missing or inaccurate prior edges may bias the inferred regulatory mechanisms. Finally, CCCvelo assumes that cells can be organized along a biologically meaningful cell-state transition process. Therefore, in practice, it should be applied to systems where a plausible continuous transition exists; otherwise, the inferred pseudotime and velocity field may reflect model-imposed ordering rather than meaningful biological dynamics.

In summary, although methods such as HoloNet [55], COMMOT [47], and NicheCompass [57] incorporate intracellular signaling information to varying degrees, either in their core models or in *post hoc* analyses, multilayer signaling analysis-based methods provide a more explicit representation of L-R-TF-TG regulatory networks in spatial CCC inference. These methods move beyond LR co-expression and offer a more mechanistic view of spatial communication. Although these methods provide a more realistic and comprehensive framework, they still primarily rely on transcript-level expression data. To bridge the gap between RNA expression and protein-, peptide-, or neurotransmitter-mediated LR signaling, as well as TF–TG regulatory relationships constrained by chromatin accessibility, future work could benefit from multi-omics analyses that integrate ST data with proteomics, metabolomics, and chromatin accessibility profiles such as ATAC-seq.

### Caveats of permutation-based cell–cell communication inference

Because there is no ground-truth annotation for CCC events, many existing methods rely on permutation-based strategies to identify putative communication patterns. This reliance spans multiple methodological paradigms: in addition to permutation-based statistical frameworks, OT-based approaches such as COMMOT [47], SPIDER [40], and SCOTIA [51] incorporate permutation-based hypothesis testing to identify cell-type- or cluster-specific, as well as spatially co-localized, LR pairs, while the contrastive learning-based method CellNEST [56] constructs negative samples using location-permuted cell graphs. Despite their differing implementations, these approaches share a common assumption, that is, the permuted data represent a “no communication” scenario, corresponding to a valid null hypothesis or appropriate negative samples. However, as we discuss below, this assumption can be violated in the presence of tissue heterogeneity and structured cell-type distributions.

#### Spatial pattern can confound permutation results

As discussed in Section Cell–cell communication inference with permutation-based hypothesis tests, cell-type- or cluster-specific permutation tests may overlook regional LR interaction patterns that arise from mixed cell types or subclusters. To address this limitation, many CCC methods adopt spatial co-localization-based permutation tests to identify LR interactions with spatial heterogeneity. However, the resulting permuted distributions and their associated null hypotheses may be mismatched depending on the underlying tissue structure.

**Figure 2:**
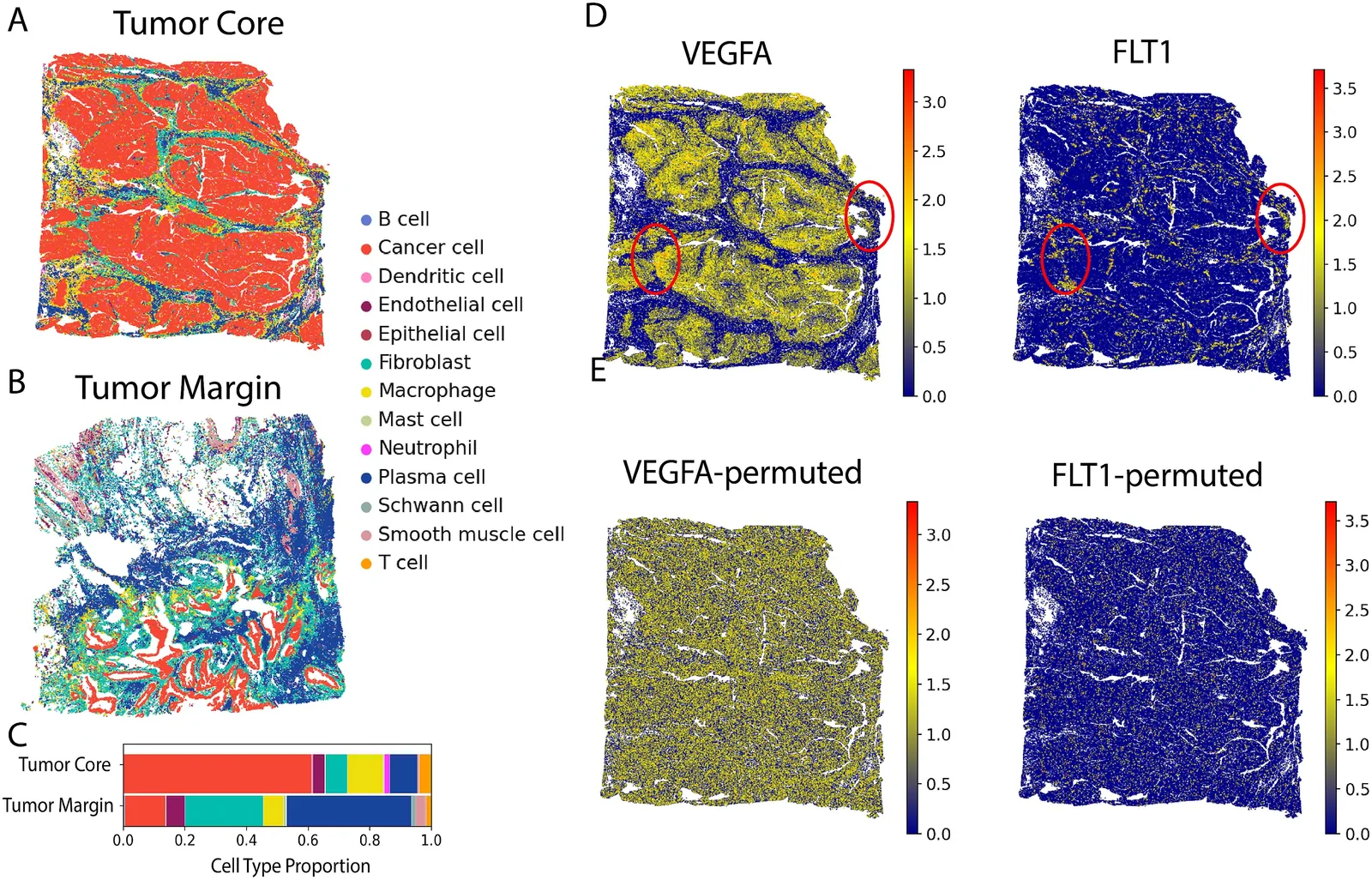
Colorectal cancer exhibits pronounced spatial heterogeneity. (A) Spatial distribution of cells in a colorectal tumor core region, where cancer cell is the dominant cell type. (B) Spatial distribution of cells in a colorectal tumor margin region, where various cell types are more evenly distributed. (C) Stacked barplot shows cell-type composition proportions of tumor core and margin regions. (D) Spatial expression patterns of ligand *VEGFA* and receptor *FLT1* in the tumor core region. (E) Spatial expression patterns of *VEGFA* and *FLT1* after random permutation of cell locations.

For example, colorectal cancer ST data often exhibit substantial spatial heterogeneity depending on the manually selected tissue region of interest, disease state, and tissue size. Tumor core regions (Fig. 2A) tend to be more homogeneous than tumor margin regions (Fig. 2B), as tumor cores are frequently dominated by cancer epithelial cells (Fig. 2C) [42]. The ligand *VEGFA* and receptor *FLT1* form a cognate LR pair whose expression patterns are spatially co-localized and highly co-expressed, particularly at the interface between cancer cells and endothelial cells (Fig. 2D). However, in such heterogeneous tissues, spatial co-localization permutation may fail to generate an appropriate null model of spatial independence between ligand and receptor expression. Because cancer cells are spatially abundant, permutation can still place nearly every *FLT1*-expressing receiver cell near cancer cells with moderate *VEGFA* expression (Fig. 2E), thereby preserving residual LR co-localization rather than fully disrupting it (Fig. 2E).

A similar concern arises for the location permutation strategy used in SCOTIA [51] (see Section Unbalanced optimal transport relaxes competition constraint), which is applied only to spatially adjacent cells, a subset of the full tissue. Such restricted cell pools are more likely to be homogeneous and may suffer from limited variability. Indeed, SCOTIA acknowledges that this strategy can lead to reduced statistical power when detecting signals in specific cell subtypes [51]. These observations highlight the need for caution when applying location permutation tests and for verifying whether the resulting permuted distributions adequately represent the desired null hypothesis for a given ST dataset.

Deep learning-based frameworks face similar concerns. CellNEST [56] employs a DGI-based contrastive learning framework to infer L-R-L-R relay communication networks, where corrupted graphs are generated by random feature permutation and contrasted against the original graph. This design assumes that corruption sufficiently disrupts structure–feature dependencies, producing a meaningful discriminative signal. However, in highly homogeneous ST tissues, original and corrupted graphs may remain similar, weakening the contrastive objective and reducing representation robustness. This could limit generalizability across tissues with different levels of heterogeneity. Therefore, sensitivity analyses evaluating graph corruption effectiveness across tissue contexts are important for reliable inference.

#### Cell-type composition can confound permutation results

A major focus of existing CCC methods is the inference of communication between cell types [2, 34, 51]. However, spatial co-localization-based permutation tests do not necessarily incorporate cell-type information. Methods such as SpatialDM [33] and SPIDER [40] adopt a two-stage strategy, first identifying spatially co-localized LR pairs and then associating these pairs with cell types to derive cell-type-specific CCC conclusions. In such pipelines, cell-type composition can act as a confounder during the initial location-based permutation step.

**Figure 3:**
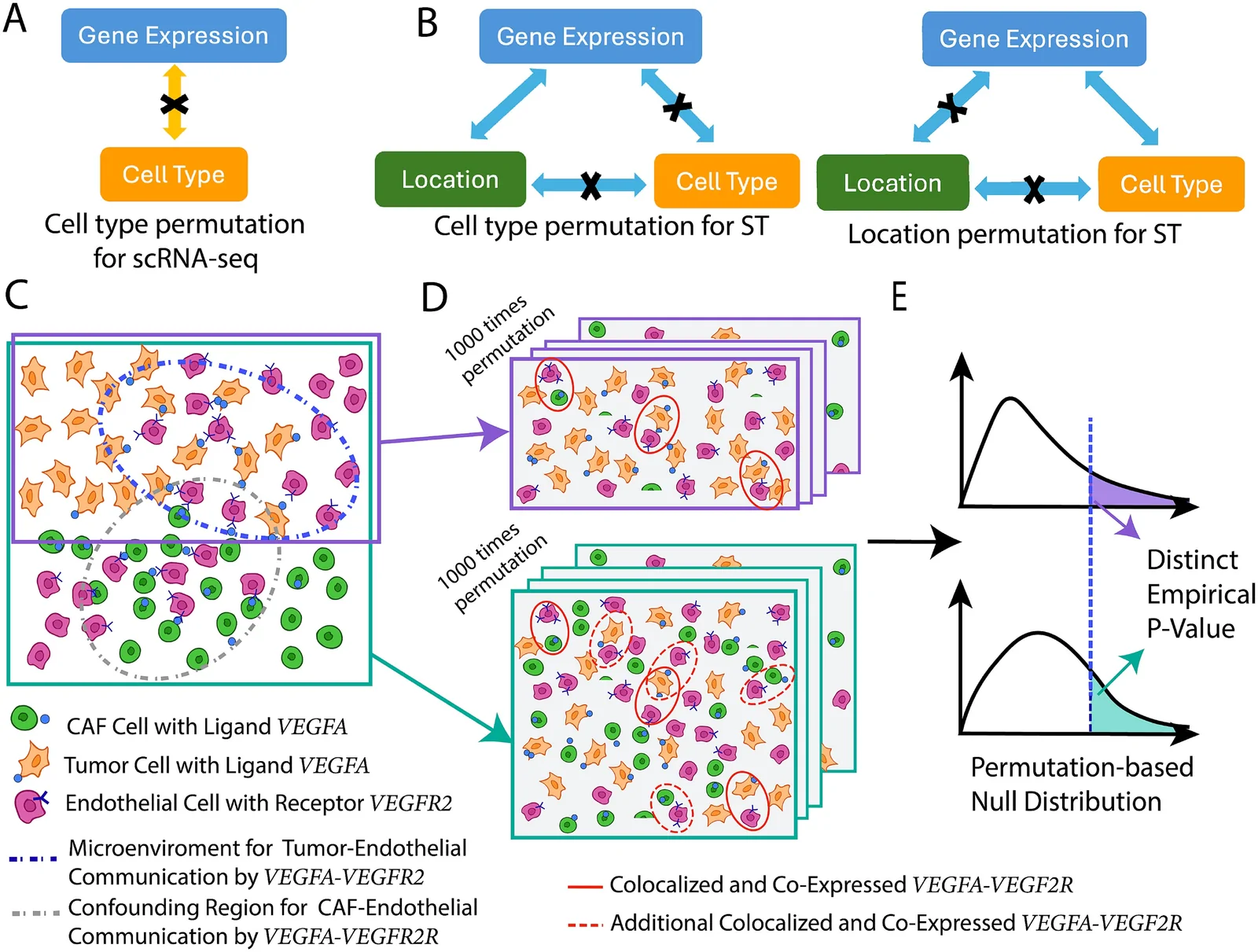
Cell-type composition confounds co-localization permutation tests. (A) Illustration of how permutations break the associations between gene expression and cell type in scRNA-seq data. (B) Illustration of how permutations break the associations among gene expression, cell location, and cell type in ST data. (C) Tumor angiogenesis microenvironment in which *VEGFA* secreted by tumor cells binds to *VEGFR2* expressed by spatially co-localized endothelial cells (top dashed outline) . A neighboring region (bottom dashed outline) exhibits *VEGFA*–*VEGFR2* interactions between CAFs and endothelial cells that also promote angiogenesis. (D) Location-permuted data showing spatially co-localized and co-expressed cells; the upper panel includes tumor-endothelial interactions, whereas the lower panel includes additional CAF-endothelial interactions. (E) Null distribution of co-localization and co-expression statistics for *VEGFA*-*VEGFR2* across 1,000 permutations. Inclusion of *VEGFA*-expressing CAFs biases the permutation-based null distribution when cell-type information is not accounted for.

In scRNA-seq-based CCC analysis, where spatial information is absent, most methods [38, 73] aggregate ligand and receptor expression at the cell-type level and permute cell-type labels (Fig. 3A), thereby breaking associations between gene expression and cell identity. In contrast, ST data preserve individual cell locations, which are explicitly utilized by recent CCC methods [34, 47]. Consequently, permuting cell-type labels cannot disrupt associations between gene expression and spatial location, and likewise, location permutation cannot eliminate associations between gene expression and cell type (Fig. 3B). As a result, whether such permutation schemes generate data consistent with the intended null distribution remains questionable.

As illustrated by the toy example in Fig. 3, when detecting tumor angiogenesis mediated by the *VEGFA*–*VEGFR2* LR pair, communication events associated with non-tumor angiogenesis in adjacent regions can reduce statistical power. Compared with analyses focusing solely on *VEGFA*–*VEGFR2* communication between tumor cells and endothelial cells (Fig. 3D, top), including cancer-associated fibroblasts (CAFs) that also express high levels of *VEGFA* (Fig. 3D, bottom) introduces additional spatially co-localized background interactions in the permuted data, leading to larger and less significant *p*-values (Fig. 3E). This example demonstrates that cell-type composition can further contribute to mismatches between permuted data and the intended null distribution.

Bayesian regression-based approaches such as Spacia [34], which explicitly identify cell-type-specific, spatially co-localized LR interactions, represent one potential alternative. In addition, functional CCC methods, including SpaTalk [74] and NicheNet [43], which prioritize LR interactions linked to downstream biological responses, offer complementary strategies for mitigating these confounding effects. Taken together with the effects of spatial heterogeneity discussed above, these observations highlight that permutation-based CCC inference in ST data can be jointly confounded by spatial organization and cell-type composition.

#### Moving beyond permutation frameworks

To mitigate the inherent limitations of permutation-based inference in complex tissue architectures, several promising directions deserve further exploration. The key challenge is not merely the use of permutation itself, but whether the permuted data correspond to a biologically meaningful and statistically valid null model. The core null hypothesis of location-permutation tests is that LR interactions do not exhibit spatial heterogeneity. However, permuting cell locations before calculating LR communication scores may create spurious spatial proximity and artificially inflate communication scores, as shown in Section Spatial pattern can confound permutation results.

One possible alternative is to decouple communication-score estimation from spatial heterogeneity testing. Specifically, LR communication scores could first be computed on the observed tissue architecture, thereby preserving the biologically observed sender–receiver neighborhoods. Spatial permutation or other resampling procedures could then be applied to the resulting communication-score field to test whether these observed scores exhibit significant spatial heterogeneity. Non-permutation-based SVG testing provides another promising direction for evaluating similar hypotheses. For example, SPIDER [40] introduces a SVI test, discussed in Section Collective optimal transport incorporates cell competition, that applies SVG-style testing to LR communication scores in order to identify spatially heterogeneous LR interactions.

Importantly, cell-type-specific test should not be treated only as a downstream analysis after location test. Incorporating sender and receiver cell types directly into the inference model would help mitigate the confounding effect of cell-type composition. Future CCC methods may benefit from a unified statistical framework that jointly accounts for cell-type-specific variation, spatial autocorrelation, and regional heterogeneity. Such a framework would allow investigators to distinguish genuine spatially organized communication from signals driven by uneven cell-type composition, local tissue structure, or residual spatial dependence in gene expression.

## Mechanism-aware cell–cell communication beyond spatial proximity

While spatial proximity, such as paracrine or juxtacrine signaling, is a fundamental prerequisite for CCC, it is far from sufficient for determining whether communication occurs. Beyond spatial constraints, the molecular determinants of LR binding critically shape whether an interaction is biochemically plausible. Likewise, signal transduction processes, including downstream intracellular signaling cascades and transcriptional responses, ultimately determine whether LR engagement leads to functional communication rather than passive co-expression. Furthermore, communication is often condition-specific, varying across biological states, disease contexts, developmental stages, or patient samples. In this section, we review how CCC methods address these 4D: (i) incorporation of LR binding mechanisms beyond simple proximity; (ii) integration of signal transduction to identify functionally active communication events; (iii) handling resolution-dependent cell-type ambiguity in ST data; and (iv) detection of condition-specific or differentially enriched communication across multiple samples.

Although many CCC inference methods incorporate biochemical constraints or downstream signaling priors, it is important to distinguish mechanistic interpretation from mechanistic inference. Most ST-based CCC analyses infer ligand and receptor activity from transcript-level expression, spatial proximity, and prior knowledge of LR interactions or signaling pathways. These features can support biologically meaningful interpretation of predicted CCC events, but they do not directly demonstrate physical signal transmission. Ligands and receptors are functional at the protein or small-molecule level, whereas ST data usually measure mRNA abundance, which may not reflect ligand secretion, receptor availability, receptor binding, post-translational modification, or downstream signaling activity. Therefore, biochemical constraints and signaling priors should be viewed as providing mechanistically informed interpretation rather than direct evidence of *in vivo* mechanistic communication. Stronger mechanistic inference requires orthogonal evidence, such as spatial proteomics, perturbation experiments, or time-resolved measurements across physical time points as validation.

### Biochemical constraints on ligand–receptor signaling

Because CCC requires LR interactions, many approaches use ligand and receptor gene expression to define potential communication events. However, the multisubunit structure of LR complexes is also essential for accurate modeling of LR interactions. For example, the interleukin-2 receptor (*IL2R*) consists of three subunits, *IL2RA*, *IL2RB*, and *IL2RG*, that together form a high-affinity IL-2 receptor complex [75]. The absence of expression of any subunit impedes inference of LR binding and subsequent communication [11]. Tools such as CellPhoneDB [38], CellChat-v2 [2], and COMMOT [47] extend simple co-expression frameworks by explicitly incorporating multimeric ligand and receptor composition. For instance, these methods define the expression level of a ligand or receptor complex using the minimum [47] or geometric mean [2] expression across subunits, ensuring that all required components are co-expressed before considering an interaction valid.

Beyond core LR binding, additional cofactors modulate communication strength. These include soluble agonists that enhance ligand binding probability, antagonists that inhibit binding, and stimulatory or inhibitory membrane-bound co-receptors (e.g. binding of ligand *MHC* to co-receptors *CD4*+*CD8* during T cell activation [76]) [73]. CellChat [2] explicitly models cofactor-mediated enhancement and inhibition effects and incorporates them into its curated database, and HoloNet [55] leverages this information. Although Spacia [34] does not explicitly model cofactor regulation, its framework can inherently accommodate multi-ligand-to-one-receptor relationships. By using multiple ligands to predict receptor expression, the sign and magnitude of ligand-specific coefficients can implicitly reflect stimulatory or inhibitory influences, thereby capturing combinatorial effects in a data-driven manner. In contrast, CellPhoneDB-v5 [77] explicitly acknowledges uncertainties in the physiological roles and context-dependent specificity of cofactors and therefore avoids encoding combinatorial effects in its curated database.

Communication efficiency is further constrained by the biophysical kinetics of LR binding [78]. Ligand diffusivity varies depending on factors such as molecular weight and the tortuosity of the extracellular space [47]. To approximate ligand diffusion, methods such as SpatialDM [33], HoloNet [55], and COMMOT [47] assume that interaction likelihood decays with increasing intercellular distance. This distance-dependent decay is incorporated as a weighting term in the computation of LR binding probability, reflecting the intuition that ligands preferentially bind nearby receptors, whereas effective signaling becomes less probable at longer distances. COMMOT applies a uniform and relatively large spatial distance limit for all LR species by default, while acknowledging that species-specific estimation of the distance parameter $T_{l,r}$ could reduce false-positive CCC links. In contrast, HoloNet [55] allows each LR species to have its own decay rate, modeled as a learnable parameter in an exponential kernel [25]. Spacia [34] further parameterizes the decay rate in a logit-based form, allowing it to vary not only across LR pairs but also across different cell-type combinations. Although identifiability of true diffusion rates remains uncertain, explicitly modeling LR-specific variation is conceptually valuable for capturing biologically meaningful differences.

### Linking cell–cell communication to downstream functional responses

Although only a subset of CCC methods explicitly model multilayer L-R-TF-TG signaling, many methods still incorporate downstream functional responses in a broader sense. These responses may include predicted target genes, pathway activities, and transcriptional modules. Here, we summarize how existing methods link inferred CCC events to downstream functional consequences beyond LR detection alone.

Many widely used CCC frameworks extend beyond LR identification to model downstream signal transduction. CellChat-v2 [2] aggregates significantly identified LR interactions into pathway-level signaling networks based on the CellChatDB database, thereby extending the interpretability of individual LR pairs to biologically coherent pathway activities. After computing the signaling strength transport matrix, COMMOT [47] associates the strength of incoming signals in receiver cells with expression changes in downstream intracellular genes, linking CCC conduction strength to transduction responses. Co-expression of correlated downstream genes and pathway markers provides a more interpretable view of functional CCC activity. Similarly, SPIDER [40] first identifies SVIs based on interface-level LR signaling strength and then filters these candidates by requiring that receptor activation profiles be significantly correlated with at least one spatially variable transduction target gene (svTF). Only SVIs supported by downstream spatial signaling in receiver cells are retained as functional interactions.

Rather than using two-step frameworks that separately model sender–receiver interaction and downstream transduction, other approaches embed downstream signaling or target gene information directly into the LR inference model. NicheNet [43], CellCall [79], and SpaTalk [74] bridge LR binding with downstream gene responses by constructing LR-target regulatory networks. NicheNet trains such networks using ligand perturbation data to quantify intracellular target gene responses and infer ligand-to-target regulatory relationships, whereas CellCall integrates TF activity to model receptor-to-target regulatory cascades. Although NicheNet and CellCall were originally developed for dissociated scRNA-seq data, several ST-based CCC frameworks, including NicheDE [37], NCEM [80], and CellNEST [56], have incorporated these models to filter LR pairs linked to specific biological functions. SpaTalk [74] further extends LR-target modeling by incorporating spatial proximity, prioritizing LR interactions whose downstream transcriptional responses are spatially coherent across tissue regions.

Recent methods further integrate intercellular conduction and intracellular transduction to model the dynamics of multicellular communication. CellNEST [56] bypasses explicit intracellular modeling and instead constructs multi-hop relay communication networks by iteratively propagating LR interactions in an L-R-L-R manner, enabling discovery of communication chains across spatially adjacent cell populations. In contrast, FlowSig [81] emphasizes intracellular modeling and adopts a graphical causal framework to link intercellular inflow, intracellular gene regulatory modules (GEMs), and outgoing ligand outflow into directed intercellular flow networks. For scRNA-seq data, FlowSig infers inflow indirectly from receptor expression and average intracellular TF activity, constraining the causal graph using perturbation-based conditional invariance. It assumes that edges in the true inflow-GEM-outflow graph remain invariant under changes in confounding covariates such as stimulation, disease, or time. In contrast, for ST data, FlowSig infers inflow directly from spatially constrained LR strength, such as the inferred amount of received ligand signal from COMMOT [47]. However, this spatially constrained inflow reflects ligand accessibility in space rather than TF-encoded signal reception and thus represents a conceptually distinct quantity from the TF-weighted inflow used in scRNA-seq.

Overall, spatially constrained co-localized communication and target-gene-mediated functional signal transduction represent complementary but not strictly equivalent biological quantities. Incorporating both types of information when identifying LR communication events can substantially reduce false-positive interactions and ensure that inferred CCC events reflect genuine downstream functional relevance.

### Resolution-dependent cell-type ambiguity in cell–cell communication inference

Though ST technology provides cell spatial location information that is absent from dissociated scRNA-seq data, a major technical challenge arises from the heterogeneous spatial resolution of different ST platforms. In subcellular or single-cell-resolution image-based technologies, such as CosMx, Xenium, or MERFISH, cell segmentation algorithms are used to assign detected transcripts to individual cells. Therefore, communication inference can be performed more directly based on true cellular neighborhoods. However, these platforms often have lower gene detection sensitivity or restricted gene panels compared with sequencing-based technologies. As a result, relevant LR pairs, downstream TFs or TGs may not be fully captured, potentially leading to biased or incomplete inference.

In contrast, spot-based technologies usually provide broader transcriptome coverage, but at the cost of reduced spatial resolution. For example, a 10*× * Visium spot is *∼ *55 *μ *m in diameter and typically contains multiple cells. Consequently, the observed expression of ligands and receptors in a single spot may originate from different cell types within the same spot. This creates ambiguity when interpreting cell-type-level communication, because the inferred sender and receiver signals may reflect aggregated spot-level expression rather than true interactions between specific cell types. We summarize how different methods address this resolution issue in Table 1.

Some methods, such as Spacia, SCOTIA, and CCCvelo, are primarily designed for single-cell or subcellular-resolution ST data and are therefore not directly applicable to conventional spot-level data without additional preprocessing. Among methods that can be adapted to spot-level data, COMMOT, stLearn, and NicheCompass either simplify each spot as a spatial unit and rely on clustering to define the cell type, or assign the dominant cell type of the upstream deconvolution to each spot. In these cases, mixed cell-type composition within a spot is not explicitly modeled, and the resulting communication scores should be interpreted as spot-level or domain-level signals rather than precise cell-type-specific interactions.

In contrast, several methods explicitly incorporate strategies to handle mixed cell types within spots. HoloNet accepts cell-type information as model input and can use either one-hot categorical cell-type labels or continuous cell-type proportion matrices derived from deconvolution. CellChat-v3 first computes a cell-type-free communication probability and then weights it using deconvoluted sender and receiver cell-type proportions to obtain cell-type-specific communication scores. SpaTalk performs cell-type deconvolution using a reference scRNA-seq atlas and then reconstructs a single-cell-resolution ST atlas by projecting scRNA-seq cells onto spatial spots. SpaceTravLR applies cell-type mapping to assign up to three likely cell identities to each spatial spot; spots with multiple high-confidence identities are split into identical pseudo-spots with the same spatial location yet distinct cell-type labels.

Overall, beyond the common simplification of assigning a dominant cell type to each spot, there is currently no standardized strategy for resolving mixed-cell composition in spot-level CCC inference. Nevertheless, cell-type assignment is a critical preprocessing step that strongly affects the interpretability of inferred communication events. With the emergence of higher-resolution technologies and reduced profiling costs, such as Xenium 5K, which measures *∼ *5000 genes, and Visium HD, which provides near single-cell-scale spatial bins of 2–8 *μ *m while retaining full transcriptome-wide coverage, more realistic CCC inference is becoming increasingly feasible. These developments also highlight the need for systematic benchmarking of CCC methods on emerging high-resolution platforms to evaluate their advantages, limitations, and robustness across spatial resolutions.

### Added value of multi-sample, condition, and temporal analyses

LR interactions can vary substantially across biological conditions, including disease severity, treatment response, and developmental stage. Beyond identifying spatially co-localized LR pairs, many CCC frameworks therefore extend their analyses to multi-sample settings to detect interactions whose signaling strength differs across conditions. A common strategy is to infer CCC separately for each sample and then apply statistical testing to compare interaction strength between conditions at the level of LR pairs or signaling pathways. For example, CellChat [2] performs per-sample inference followed by permutation-based differential testing at both the LR and pathway levels; SCOTIA [51] applies either Mann–Whitney U-tests or mixed-effects models, treating sample identity as a random effect and treatment as a fixed effect; and FlowSig [81] uses Wilcoxon rank-sum tests to detect differential inflow across conditions, where inflow in ST data is estimated from spatially constrained LR signaling. Beyond differential LR testing, methods such as MultiNicheNet [82] and the scRNA-seq version of FlowSig further integrate downstream signal transduction, including TF activation and target gene responses, to identify ligands or inflow associated with distinct functional states across conditions.

Spatiotemporal transcriptomics extends ST by introducing a temporal axis, which may represent discrete physical time points, such as embryonic developmental stages [83], or biological pseudotime that captures processes such as tumor progression [84]. These datasets enable reconstruction of spatially resolved transcriptional dynamics across multiple samples or time points. However, pairwise differential testing captures only local contrasts and does not model the global structure of CCC dynamics across multiple conditions or temporal contexts. Tensor-cell2cell [85] addresses this limitation by jointly decomposing LR communication patterns from multiple samples using a tensor-based framework. In its original formulation, Tensor-cell2cell relies on cell-type-level LR communication inferred from scRNA-seq, yielding a tensor with dimensions corresponding to samples, LR pairs, sender cell types, and receiver cell types. How spatial proximity constraints and spatial heterogeneity can be integrated into such tensor representations remains an open question.

Several scRNA-seq methods have also leveraged temporal information to infer dynamic CCC. TraSig [86] identifies LR pairs with coherent expression dynamics along pseudotime, whereas TimeTalk [87] focuses on LR pairs exhibiting coordinated expression across discrete physical time points and can reveal stage-specific signaling programs. However, because scRNA-seq lacks spatial information, these approaches capture intrinsic temporal coherence without ensuring spatial proximity between interacting cells. Nonetheless, temporally regulated LR interactions remain biologically informative and, when supported by spatial data, may reveal communication programs that actively drive disease progression and support patient stratification. Together, these observations highlight an important opportunity to integrate spatial constraints with temporal dynamics for characterizing CCC in spatiotemporal transcriptomic data. Developing such frameworks represents a promising direction for deciphering context-dependent communication programs across both space and time.

## Evaluation and benchmarking frameworks for cell–cell communication inference

In most ST data, true CCC events are not directly measured. Due to the unobservable dynamics of LR binding, signal transduction, and downstream transcriptional activation, CCC inference lacks a well-established gold standard. This makes it difficult to rigorously evaluate model performance and leads to substantial variability across different methods. Existing benchmark studies, such as ESICCC [88] and Dimitrov *et al*. [89], mainly evaluate CCC inference methods for scRNA-seq data, although some of their evaluation strategies can be extended to spatial CCC methods. Here, we summarize existing evaluation strategies into three categories: simulation-based benchmarking, computational benchmarks with biological assumptions, and indirect biological benchmarks.

### Simulation-based benchmarking

Simulation-based benchmarking generates synthetic data with predefined communication structures and then evaluates whether a method can recover the simulated LR or LR–TG relationships. In principle, simulation provides explicit ground truth and allows systematic control of spatial pattern, ligand diffusion, noise level, and cell-type composition. However, the validity of such benchmarks strongly depends on whether the simulator reflects realistic CCC mechanisms. Existing simulation benchmarks can be roughly divided according to the synthetic mechanism being tested.

First, some methods simulate spatially constrained LR interaction. For example, SpatialDM [33] generates positive-interaction and no-interaction simulations to evaluate true positive and false positive rates of spatially co-expressed LR detection. On the other hand, COMMOT [47] constructs simulations with predefined ligand and receptor spatial distributions, including cases where multiple ligands compete for the same receptor, to test whether OT-based inference can recover spatial communication patterns [47].

Second, some simulations focus on ligand diffusion or distance decay. For example, stMLnet [67] simulates ligand diffusion using a PDE-derived inverse-distance weighting scheme and evaluates whether the model can recover both LR interactions and downstream target genes.

Third, some simulations evaluate downstream or higher-order signaling structure. HoloNet [55] constructs simulated data to test whether inferred LR rankings are consistent with spatial information and known gene regulatory networks. CellNEST [56] designs relay-network simulations in which a sender cell transmits a signal to an intermediate receiver, which then relays another signal to a third cell. Tensor-cell2cell [85] uses simulations to test whether tensor decomposition can recover context-dependent changes in sender cells, receiver cells, and LR pairs.

Fourth, some simulations are designed for broader spatial or niche-level effects rather than single LR recovery. MISTy [36] uses synthetic spatial systems to evaluate whether multiview models can recover intrinsic, local, and broader tissue-level dependencies. Niche-LR/Niche-DE [37] benchmarks context-dependent niche effects on simulated CosMx, Slide-seq, and Xenium-like data. NicheCompass [57] is mainly designed to benchmark niche recovery, gene program inference, and batch-effect removal.

In summary, a primary challenge of simulation-based benchmarking is that different CCC methods are built upon distinct mechanistic assumptions. Consequently, no single data simulation strategy can comprehensively or fairly evaluate all methods. While simulated data remain valid for assessing model efficacy within its own structural framework, it primarily tests whether a method can recover its specific synthetic mechanism. Therefore, simulation-based evaluations should be interpreted as assumption-dependent benchmarks rather than unbiased, universal gold standards.

### Computational benchmarks with biological assumptions

In the absence of experimentally measured ground truth CCC events, several computational benchmarks evaluate CCC inference methods based on simplified biological assumptions. These benchmarks do not directly observe true LR binding events, but instead test whether predicted communications are consistent with expected biological properties, such as spatially constrained communication, downstream cytokine activation, or robustness to sampling variation.

#### Evaluation on ligand–receptor detection accuracy and stability

We summarized three complementary metrics to evaluate CCC inference methods. The overall stability score *S* measures the robustness of inferred CCC results and is broadly applicable across CCC inference methods. DLRC is designed specifically for spatial CCC inference methods that can detect coexpressed LR pairs, such as COMMOT [47], SpatialDM [33], and CellChat [2] (see Supplementary Table S1 for method details). Because DLRC is based on the assumption of local LR coexpression, it is most appropriate for methods that model short-distance communication, including paracrine and juxtacrine signaling, and is not intended for long-distance communication methods such as CLRIA [50]. Finally, for cytokine ligands, the cytokine-activity-supported odds-ratio metric, $OR_{\textrm{topK}}$, can assess the biological reliability of the LR pair for any CCC inference methods.

***Robustness to cell subsampling***

This metric assesses the robustness of CCC inference methods under moderate changes of the input data. ESICCC [88] evaluates stability by subsampling cells at different proportions and comparing the LR pairs predicted from subsampled data with those predicted from the original full data. The overlap is measured by the Jaccard index. The overall stability score *S* is defined as the aggregation of Jaccard indices across different subsampling levels:


(1) \begin{align*}& S =\frac{1}{ \sum_{i} (1-J_{i}) }, \quad J_{i} =\frac{ |LR_{\textrm{sampled},i} \cap LR_{\textrm{original}}| }{ |LR_{\textrm{sampled},i} \cup LR_{\textrm{original}}| }\end{align*}

where $LR_{\textrm{sampled},i}$ is the set of LR pairs predicted from the *i*th subsampled dataset, and $LR_{\textrm{original}}$ is the set of LR pairs predicted from the full dataset. A higher $J_{i}$ indicates greater overlap with the original prediction at a given subsampling level, and a larger *S* indicates more consistent LR predictions across different subsampling levels.

***Spatially constrained LR interaction***

One commonly used assumption is that spatially closer cells are more likely to communicate. Based on this assumption, ESICCC [88] and stMLnet [67] evaluate whether predicted LR pairs show stronger expression association between nearby sender–receiver cell pairs than between distant cell pairs. This is measured by the differential LR correlation (DLRC) metric. A higher DLRC score indicates that the predicted LR pairs are more consistent with the spatial-proximity assumption.

For a predicted LR pair, all possible sender–receiver cell pairs are first ranked by their Euclidean distance and then are defined as close(rank *≤ *  *h*) or distant(rank > *h*) groups. h is different level of distance proportions, such as *10%*, *20%*, *30%*, or *40%*. For each group, the association between ligand expression in sender cells and receptor expression in receiver cells is quantified using mutual information MI. The overall DLRC score can be summarized across different distance proportions:


(2) \begin{align*} {\textrm{DLRC}} &= \sum_{h \in H} (0.5-h) \cdot I(p_{h} < 0.05) \cdot \Delta_{h}, \nonumber \\ \Delta_{h} &= \operatorname{median} \left( MI^{\textrm{close}}_{h} \right) - \operatorname{median} \left( MI^{\textrm{distant}}_{h} \right),\nonumber \\ MI_{h}^{c} &= \sum_{l,r \in N_{c}(h)} p(l,r) \log \frac{p(l,r)}{p(l)p(r)}.\end{align*}

where *H* is the set of distance proportions, $p_{h}$ is the Wilcoxon test *p*-value at proportion *h*, and $I(p_{h} < 0.05)$ is an indicator function. c $\in \{\text{close, distant}\}$, $N_{c}(h)$ denotes close or distant cell group at proportion *h*, *p(l,r)* is the joint probability distribution of ligand expression *l* and receptor expression *r*, and *p(l)* and *p(r)* are their marginal distributions.

***Cytokine activity-supported LR interaction***

Another biological assumption is that functional cytokine-mediated communication should induce downstream transcriptional activity in the receiver cell type. Based on this assumption, Dimitrov *et al*. [89] used 43 cytokine expression signatures from CytoSig [90] to infer cytokine activity for each cell type. They then evaluated whether top-ranked cytokine-mediated CCC predictions were enriched for cytokines with positive downstream activity in the corresponding receiver cell type. This enrichment was quantified using odds ratios derived from Fisher’s exact test across different ranked prediction ranges:


(3) \begin{align*}& {\textrm{OR}}_{\textrm{topK}} = \frac{a/b}{c/d} =\frac{ad}{bc}\end{align*}

where *a* is the number of top-ranked cytokine-mediated predictions whose cytokine ligand is active in the receiver cell type, *b* is the number of top-ranked predictions without corresponding cytokine activity, *c* is the number of background predictions with corresponding cytokine activity, and *d* is the number of background predictions without corresponding cytokine activity. ${\textrm{OR}}>1$ indicates that topK-ranked CCC predictions are enriched for cytokines with downstream activity in receiver cells.

#### Evaluation on cell-type-specific ligand–receptor matching accuracy and stability

Many CCC inference methods, such as CellChat [2] and Spacia [34], focus on cell-type-specific LR interactions. To evaluate whether inferred LR interactions are assigned to biologically plausible sender and receiver cell types, we summarize two complementary metrics: AUROC/AUPRC for agreement with independent cell-type-specific molecular evidence and Jaccard index for robustness to cell-type annotation errors. These metrics can be broadly applied to any CCC inference methods.

***Agreement between LR to corresponding sender and receiver cell types*** This metric is based on the assumption that, for an inferred cell-type-specific interaction *(s,r,L,R)*, ligand *L* should be generally highly expressed in sender cell type *s*, and the receptor *R* should also be generally highly expressed in receiver cell type *r*, even beyond the specific ST data of interested. Therefore, independent external transcriptomic or protein-level evidence can be used for evaluation.

For instance, ESICCC [88] uses both FANTOM5 [91] cap analysis of gene expression (CAGE) profiles across 144 primary human cell types [92, 93] and proteomics data from 28 primary human hematopoietic cell populations [94] for evaluation. Similarly, Dimitrov *et al*. [89] use seven CITE-seq datasets as protein-level evidence, mainly focusing on receptor-side validation. CAGE provides relatively stable cell-type-specific transcriptomic evidence and is less affected by dropout, while proteomics and CITE-seq data provide protein-level evidence that is closer to the functional molecular form of LR communication. Therefore, these external datasets can serve as pseudo-ground-truth evidence for evaluating whether predicted LR interactions are assigned to biologically plausible sender and receiver cell types.

A predicted interaction *(s,r,L,R)* or *(r,R)* is labeled as positive if the expression of ligand *L* in the sender cell type *s* and receptor *R* in the receiver cell type *r* are both larger than a predefined threshold; otherwise, it is labeled as negative. Method performance is then evaluated using AUROC and AUPRC, where AUPRC is more appropriate when the positive and negative classes are imbalanced. Higher AUROC/AUPRC indicates better agreement between predicted CCC interactions and independent cell-type-specific expression evidence.


(4) \begin{align*}& \begin{aligned} \textrm{Precision} &= \frac{TP}{TP+FP}, \quad \textrm{Recall} = \frac{TP}{TP+FN}, \\ \textrm{AUPRC} &= \int \textrm{Precision}(\textrm{Recall})\,d\textrm{Recall}. \end{aligned}\end{align*}

***Robustness to cell annotation error***

This benchmarking evaluates the robustness to cell-type annotation errors by randomly reshuffling a fraction of cell labels and measuring whether top-ranked CCC predictions were preserved [89]. This benchmark tests the sensitivity of CCC methods to upstream preprocessing and cell-type annotation quality. Their results suggest that incorrect cell-type annotation has a stronger effect than simple cell subsampling, highlighting that reliable CCC inference depends critically on accurate cell identity assignment. This benchmark can be summarized as:


(5) \begin{align*}& J_{\mathrm{label-noise}} = \frac{ |LR_{\textrm{perturbed label}} \cap LR_{\textrm{original}}| }{ |LR_{\textrm{perturbed label}} \cup LR_{\textrm{original}}| },\end{align*}

where a higher value indicates that predictions are more stable under cell-label perturbation.

#### Evaluation on signaling transduction

Some CCC methods go beyond LR inference and predict downstream target genes regulated by ligand or receptor signals. The AUCROC/AUPRC metric for LR–TG evaluation can be applied to CCC inference methods who are capable of predict downstream genes, such as stMLnet [67], MISTy [36], HoloNet [55], and COMMOT [47]. The confidence score metric is specifically designed for multi-hop inference method such as CellNEST [56].

***LR–TG prediction accuracy*** For LR-TG evaluation, cell-line perturbation datasets are commonly assumed to be pseudo-ground truth, where specific ligands or receptors are perturbed and differentially expressed genes (DEGs) after perturbation are treated as downstream target labels. A commonly evaluation dataset contains 15 perturbed cell lines, spanning conditions such as breast cancer, ductal carcinoma *in situ*, and glioma [67].

If a method predicts that an LR pair regulates a target gene *TG*, and *TG* is a DEG after the corresponding ligand or receptor perturbation, then the predicted LR–TG link is labeled as true; otherwise, it is labeled as false. The LR–TG regulatory score is then compared with these binary labels using AUROC and AUPRC. Because LR–TG prediction is highly imbalanced, with many more negative than positive target genes, AUPRC is usually more appropriate than AUROC. A higher AUPRC indicates that the method better prioritizes true perturbation-responsive target genes.

***Multi-hop relay network confidence scoring***

CellNEST further evaluates predicted relay networks by assigning confidence scores based on independent prior evidence. It integrates PPI evidence from STRING and NicheNet, as well as TF–TG evidence from DoRothEA, to score the plausibility of predicted relay paths [56]. The logic is that a predicted relay network is more credible if its intermediate signaling and transcriptional links are supported by external curated databases. A generic relay confidence score can be written as:


\begin{align*} & \textrm{Conf}(\mathcal{P}) = \prod_{e \in \mathcal{P}} w_{e} \quad \textrm{or} \quad \textrm{Conf}(\mathcal{P}) = \frac{1}{|\mathcal{P}|} \sum_{e \in \mathcal{P}} w_{e}, \end{align*}

where $\mathcal{P}$ is a predicted relay path, and $w_{e}$ is the evidence weight of edge *e* from external databases. The predicted relay networks can then be compared with random or permuted networks. If predicted relay paths have significantly higher confidence scores than random paths, this supports their biological plausibility. This benchmark does not prove that the relay occurs *in vivo*, but it tests whether the inferred multicellular signaling paths are consistent with independent molecular interaction knowledge.

In summary, although computational benchmarking metrics provide quantitative evaluation of method performance, their core limitation is that they cannot capture the full complexity of *in vivo* tissue environments. Most of these benchmarks rely on simplified biological assumptions, such as spatial proximity, downstream pathway activation, or agreement with external transcriptomic or protein-level data. These assumptions are useful for constructing measurable pseudo-ground truth, but they only approximate selected aspects of CCC. Different metrics also favor different classes of methods: distance-based metrics may favor spatial-proximity models, downstream target benchmarks may favor methods with prior signaling networks, and stability metrics may favor conservative methods that predict fewer interactions. Therefore, computational benchmarks are valuable for systematic comparison, but their results should be interpreted as assumption-dependent evidence rather than definitive validation of true CCC events.

### Indirect biological benchmarks

Indirect biological benchmarks refer to descriptive or qualitative biological validation without defining a quantitative pseudo-ground-truth metric. These benchmarks ask whether inferred CCC patterns are consistent with known tissue anatomy, canonical signaling biology, disease-associated niches, or experimentally observed spatial organization. Unlike computational benchmarks with biological assumptions, they do not compute formal performance metrics against external biological evidence.

For example, COMMOT [47] qualitatively examines whether inferred communication directions and spatial signaling patterns match known tissue organization across ST datasets. SpatialDM [33] illustrates selected spatially co-expressed LR pairs by showing that they localize to biologically meaningful tissue regions. SpaTalk [74] illustrates inferred LR–TG s signaling networks in STARmap and seqFISH+ datasets and interprets them in relation to known spatial tissue organization. NicheCompass [57] uses inferred communication programs to characterize known anatomical or disease-associated niches, such as developmental tissue regions or tumor microenvironment niches. CCCvelo [68] provides descriptive support by examining whether dynamic CCC patterns are consistent with inferred cell-state transition trajectories.

However, descriptive indirect biological benchmarks should be interpreted cautiously. They can show that inferred CCC patterns are biologically plausible, but they do not define true CCC events or provide quantitative measures of sensitivity, specificity, or false discovery rate. Because these validations often focus on selected examples, they may overemphasize interpretable canonical pathways while leaving most predictions unevaluated. Moreover, spatial colocalization or consistency with known tissue organization does not prove functional LR signaling, since similar patterns can be driven by cell-type composition, spatial autocorrelation, or shared transcriptional states. Therefore, these benchmarks are useful for biological interpretation but are not sufficient for rigorous method comparison.

## Databases for cell–cell communication analysis

In real CCC analyses, database choice directly determines which interactions can be discovered, ranked, or excluded. Therefore, database selection should be guided by the analytical goal, tissue context, species, assay resolution, and the trade-off between coverage and specificity. Therefore, we summarized commonly used databases supporting CCC modeling, grouped by LR interactions, signaling pathways, TF-target regulation, and integrated multilayer databases; the details are summarized in Table 2.

**Table 2:** Key database resources supporting computational CCC modeling. Databases are grouped by their roles in LR interactions, intracellular signaling pathways, and TF-target regulatory networks

Category	Database	Main scope	Update status	Main caveats
LR interaction	FANTOM5	Expression-based LR map across human primary cells and tissues.	Static release	Biased toward primary cells; misses recent or context-specific LR pairs
	connectomeDB	Manually curated LR pairs for CCC analysis.	Periodically updated	Literature-curation bias; coverage depends on currently curated LR evidence
	CellPhoneDB-v5	Curated LR pairs with multimeric LR complexes.	Actively maintained	Biased toward human/mouse curated interactions.
	CellChatDB-v2	LR pairs with pathway, cofactor, co-receptor, and non-protein signaling annotations.	Versioned with CellChat paper	Complex sub-components poorly map to limited-panel ST data
	NeuronChatDB	Neurotransmitter, neuropeptide, gap junction, and synaptic signaling interactions.	Versioned with NeuronChat paper	Incomplete outside neural signaling and may miss curated entries
Signaling pathway	KEGG	Broad pathway and organism-level knowledge base.	Periodically updated	Coarse pathway boundaries lack granular transduction details
	Reactome	Curated reaction-level pathways and molecular events.	Periodically updated	Human-centric; non-human species rely on orthology inference
	SIGNOR	Directed causal signaling interactions with activation/inhibition annotations.	Periodically updated	Literature-curated; biased toward well-studied signaling proteins/pathways
TF–TG regulation	TRRUST-v2	Literature-curated TF-TG regulation with activation/repression labels.	Static release	Literature bias toward celebrity TFs; lacks tissue specificity
	DoRothEA	TF regulons with confidence levels from multiple evidence sources.	Version depends on package release	Context-independent regulons with potential indirect targets
Integrated LR–TF–TG multilayer regulation	NicheNet	LR, signaling, and gene regulatory priors linking ligands to target genes.	Actively maintained	Biased toward well-studied ligands and available perturbation data
	OmniPath	Meta-resource integrating interactions, complexes, annotations, and CCC resources.	Periodically updated	Inherits multi-source inconsistencies; requires strict filtering
	stMLnetDB	LR, R-TF, and TF–TG cascades for multilayer CCC modeling.	Versioned with stMLnet paper	Inherits biases from curated LR/TF databases

For methods focused on LR detection, curated LR interaction databases are usually sufficient and provide interpretable candidate interactions. If the goal is to identify potential novel LR pairs beyond existing curated resources, multi-omics- or sequence-informed approaches such as CeLLetter [95] may provide broader candidate coverage, although such expanded candidates usually require additional biological validation. For analyses that aim to incorporate intracellular signal transduction, pathway and TF–TG regulatory databases are needed to connect receptor activation with downstream transcriptional responses. For unified CCC frameworks that jointly model extracellular communication, intracellular signaling, and gene regulation, integrated multilayer resources provide a more direct prior-knowledge structure.

Core CCC analyses rely on curated LR interaction resources such as FANTOM5 [91], CellPhoneDB-v5 [77], and CellChatDB-v2 [2]. FANTOM5 compiles 1894 LR interactions supported by primary literature, along with an additional 528 putative interactions lacking direct experimental evidence. CellPhoneDB-v5 provides 2911 manually curated interactions and includes detailed annotations of multimeric ligand and receptor complexes. CellChatDB-v2 contains *∼ *3300 interactions and further incorporates soluble and membrane-bound stimulatory and inhibitory cofactors and co-receptors, together with pathway-level functional annotations for each LR pair. In addition to protein-based LR interactions, NeuronChatDB [96] catalogs neurotransmitter–receptor interactions underlying neuron-to-neuron communication, and these curated interactions are incorporated into the updated CellChatDB-v2.

However, literature-curated LR databases can be incomplete, potentially missing less-studied tissues or non-canonical signaling interactions. Conversely, they may also be overly broad, including L-R pairs that are implausible in the target tissue or cell types. Methods such as CellDialog [97] and CellGDnG [98] leverage ligand and receptor protein sequences together with scRNA-seq data to expand the coverage of curated LR databases while retaining only tissue- and cell-type-relevant LR pairs. Furthermore, CeLLetter [95] combines state-of-the-art protein large language models to extract protein features and incorporates receiver-cell TF activities to define LR communication scores. Together, these methods move LR databases beyond static lists of curated interactions toward context-specific, confidence-scored LR resources.

To characterize intracellular signal transduction following receptor activation, pathway databases, such as KEGG [99], Reactome [100], and SIGNOR [101] provide curated signaling pathways describing how signals propagate through intracellular protein networks. KEGG summarizes well-established signaling routes, Reactome offers a detailed reaction-level representation of molecular events, and SIGNOR focuses on causal and directed interactions indicating activation or inhibition. Collectively, these resources support reconstruction of LR-signaling-TF cascades that connect extracellular communication events to downstream transcriptional responses.

Complementing pathway databases, resources including TRRUST-v2 [102] and DoRothEA [103] curate TF–target regulatory relationships linking intracellular signaling to gene expression programs. TRRUST-v2 provides high-confidence TF–target pairs derived from literature text mining followed by manual curation, with explicit annotations of activation or repression. DoRothEA compiles TF regulons from multiple evidence sources, including literature, motif predictions, and perturbation experiments.

While many databases focus on individual components of CCC inference pipeline, NicheNet [43], OmniPath [104], and stMLnetDB [67] distinguish themselves by integrating LR interactions, intracellular signaling networks, and TF–TG regulation from diverse resources into unified multi-layer molecular interaction networks. Although these resources frequently cross-reference each other across version updates, NicheNet emphasizes L-TG regulation to prioritize ligands that likely drive specific downstream expression changes; stMLnetDB explicitly enforces signal continuity across the L-R-TF-TG axis to model the integrity of information flow; and OmniPath serves as a universal meta-resource that provides a comprehensive map of molecular interactions beyond directed regulatory paths.

## Practical guidance for choosing cell–cell communication methods

**Figure 4:**
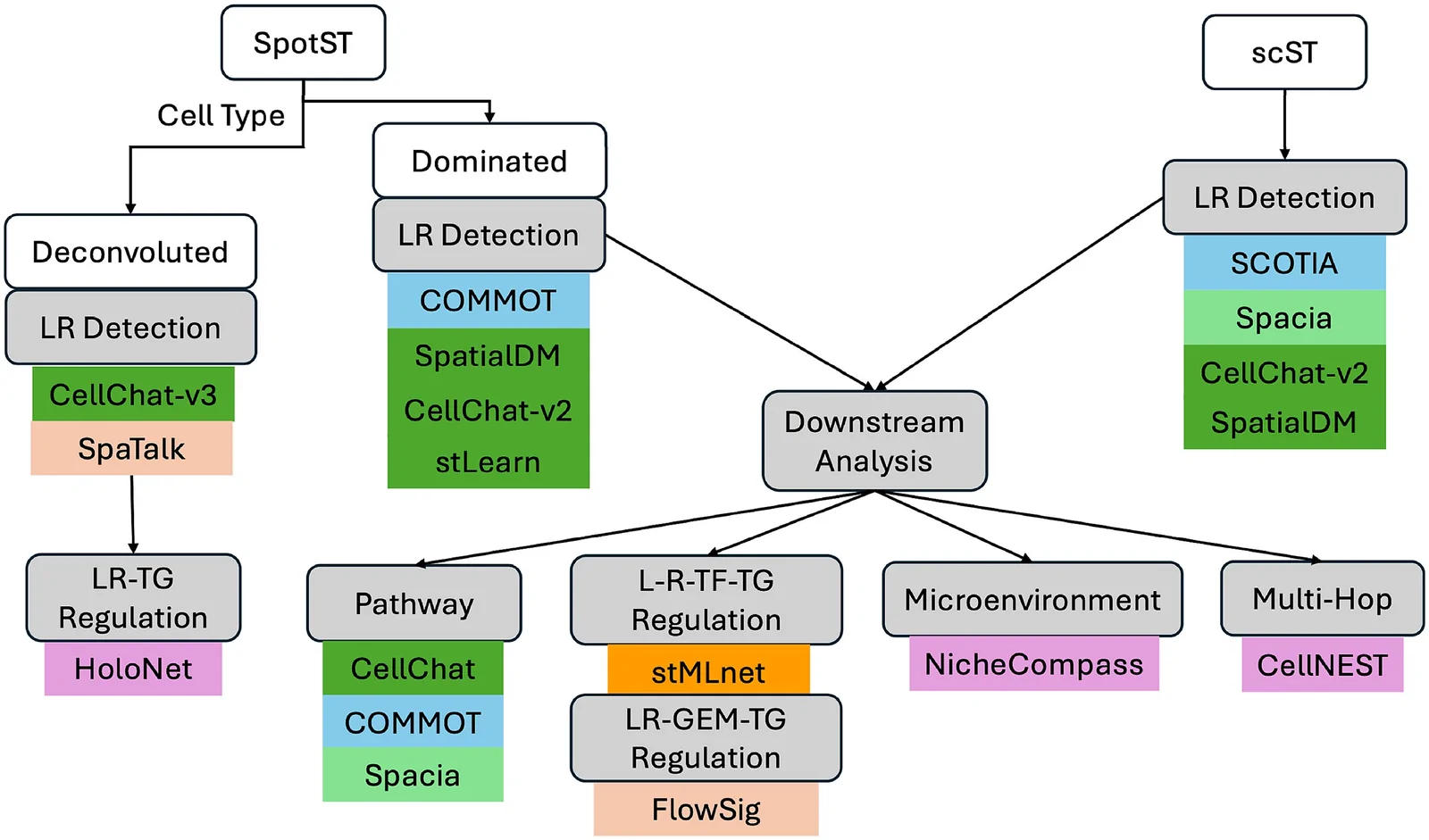
Practical selection strategy for spatial CCC methods. The flowchart summarizes representative method choices according to ST resolution, cell-type annotation strategy, and downstream analysis objective. Spot-based ST (SpotST) methods are grouped by whether they use deconvolution-derived cell-type information or dominant cell-type labels, whereas single/sub-cellular level ST (scST) methods typically use cell-level cell-type annotations. Downstream methods are organized by analytical goal, including pathway-level analysis, LR–TF–TG regulation, communication-defined microenvironment analysis, and multi-hop relay analysis.

No single CCC framework is universally optimal, and method selection should be guided by the spatial resolution, cell-type annotation strategy, gene coverage, biological question, scalability, and availability of prior knowledge. Figure 4 summarizes a practical selection strategy for representative spatial CCC methods, while Table 1 and Supplementary Table S1 provide detailed comparisons of input requirements, modeling assumptions, database usage, and output types.

For spot-based ST data, methods that incorporate deconvolution-derived cell-type proportions or reconstructed cell-level representations are generally preferable when cell-type-specific interpretation is desired. When only dominant cell-type labels are used, inferred CCC should be interpreted as spot-level or spatial-domain-level communication rather than definitive cell-level sender–receiver interactions. For single-cell or subcellular ST data, methods that directly model local cell neighborhoods are better suited for resolving cell-level communication, although scalability should be considered for very large datasets.

Dataset coverage should also guide method selection. LR-focused methods may be more robust for low-coverage or targeted-panel datasets, whereas multilayer methods that require receptors, TFs, and target genes are better supported by high-coverage datasets. Analytical goals further determine method choice: statistical inference-based methods are suitable for hypothesis-driven LR detection, OT-based approaches are useful for many-to-many communication and L-R competition, deep learning approaches are useful for communication-defined microenvironment or relay-pattern discovery, and multilayer methods are preferable when linking intercellular LR signaling to downstream intracellular signal transduction is central. Despite these practical distinctions, systematic benchmarking across tissues, spatial resolutions, and biological contexts remains limited. Therefore, method selection should be interpreted as context-dependent guidance rather than a fixed ranking, and standardized evaluation frameworks are still needed for robust CCC inference in ST data.

## Discussion

In this review, we focus on CCC methods developed specifically for ST data that explicitly leverage spatial information. Although many CCC frameworks have been developed for scRNA-seq data, CCC events inherently require co-localized sender and receiver cells with co-expressed cognate LR pairs. The additional spatial context provided by ST data is therefore essential for accurately characterizing intercellular communication. We classify 16 ST-based CCC methods into four computational paradigms: statistical inference-based, OT-based, deep learning-based, and multilayer analysis-based approaches, with particular emphasis on how each paradigm incorporates spatial information. Although permutation strategies are widely used across all three categories, we highlight important caveats, including how tissue heterogeneity and cell-type composition can confound their performance. In addition, we summarize eight complementary CCC approaches that emphasize non-spatial dimensions, such as LR biochemical mechanisms, functional communication, and shared or differential communication across biological conditions or time points. We further review databases that provide prior knowledge of intercellular conduction and intracellular signal transduction, particularly those capturing LR–TF–target gene regulatory cascades.

Awareness of tissue context is critical when selecting CCC methods, as tissue regions captured by ST experiments are manually defined and can vary substantially in size, region of interest, disease state, and cellular composition. Methods such as CellChat-v2/3 [2], COMMOT [47], and CellNEST tend to produce global conclusions by evaluating LR co-localization and co-expression across entire tissue sections, even though spatial proximity inherently constrains feasible communication patterns. In contrast, approaches such as Spacia [34] and SCOTIA [51] perform microenvironment-specific CCC analyses by evaluating LR interactions within localized, intra-cell-type spatial neighborhoods. However, as demonstrated in Section Caveats of permutation-based cell–cell communication inference, permutation-based inference strategies may amplify confounding effects introduced by tissue context, potentially biasing both global and microenvironment-specific conclusions. Identifying communication patterns that are shared across biological replicates can help mitigate context-specific artifacts. Developing statistical frameworks that explicitly model multi-tissue replicates and reduce reliance on permutation-based inference represents an important direction for future methodological development.

Current CCC pipelines typically involve identifying spatially co-localized and co-expressed LR pairs, filtering for functional interactions supported by coherent downstream signaling cascades and TF activation, and detecting differential LR activity across disease states or time points. Although approaches such as PathFinder [105] link signaling pathways to phenotypic outcomes in scRNA-seq data, a substantial gap remains in extending such frameworks to ST data. In particular, integrating spatially resolved LR interactions with their downstream signaling pathways to explain phenotypic variation and clinical outcomes remains an open challenge. In addition, *in silico* gene perturbation frameworks may further elucidate how gene regulatory mechanisms influence phenotypes and disease progression. With rapid advances in ST technologies, the field is now generating increasingly large collections of high-resolution samples with expanded gene panels and multiple biological replicates across diverse conditions. These developments underscore the need for more advanced and integrative computational tools to further dissect CCC and to enable deeper biological interpretation in spatially resolved contexts.

**Key Points**

- Spatial transcriptomics enables mechanistically grounded inference of cell–cell communication (CCC) by preserving tissue architecture and cellular proximity, overcoming limitations of scRNA-seq-based approaches.
- Current CCC methods can be organized into three major computational paradigms: statistical inference, optimal transport, and deep learning, each with distinct modeling assumptions and trade-offs.
- Spatial CCC inference introduces unique statistical challenges, including null model misspecification and confounding by tissue heterogeneity and cell-type composition.
- Integrating biochemical constraints and downstream signal transduction improves functional interpretability beyond simple ligand–receptor co-expression.
- Future CCC modeling requires uncertainty-aware, spatially explicit, and reproducible frameworks to fully leverage high-resolution spatial omics technologies.

## Supplementary Material

Supplemental_Table_1_bbag477

## Data Availability

No new data were generated or analyzed in support of this research.

## Abbreviations and terminology

**ST** - Spatial transcriptomics, capturing gene expression together with spatial coordinates at single-cell or multi-cell resolution.

**Spot** - A spatial capture unit in ST platforms that may contain one or multiple cells, depending on the technology and resolution.

**CCI** - Cell-cell interaction; an umbrella term encompassing all intercellular interaction modes, including chemical and non-chemical communication processes.

**CCC** - Cell-cell communication; LR-mediated chemical signaling and downstream intracellular signal transduction.

**Relay communication** - Multicellular communication in which signals are passed sequentially along a chain of cells.

**Sender cell** - A cell expressing ligands that initiate intercellular signaling.

**Receiver cell** - A cell expressing cognate receptors that receive intercellular signaling.

**Microenvironment** - A spatially localized cellular context characterized by specific cell compositions, spatial organization, and communication programs.

**LR / LRI** - Ligand-receptor / ligand-receptor interaction.

**LR pair** - A specific ligand-receptor combination considered as a candidate unit of intercellular communication.

**LR signaling strength** - A quantitative measure of inferred communication activity for a ligand-receptor pair between sender and receiver cells, defined by method-specific models.

**TF** - Transcription factor involved in downstream regulatory responses.

**TG** - Target genes regulated by transcription factors.

**Signal Conduction** - Intercellular LR signal transmission between sender and receiver cells.

**Signal Transduction** - Intracellular downstream signaling within receiver cells following receptor activation, including R-TF-TG regulatory relationships and pathway-level responses.

**GEM** - Gene expression module used in FlowSig [81] to represent intracellular transcriptional programs.

**SVI** - Spatially variable interaction defined in SPIDER [40]; ligand-receptor pairs exhibiting spatially heterogeneous interaction patterns across tissue interfaces.

**OT** - Optimal transport; a mathematical framework for inferring communication flows between sending and receiving cells.

**COT** - Collective optimal transport (COMMOT), modeling competition among multiple ligand-receptor interactions.

**UOT** - Unbalanced optimal transport (CLRIA, SCOTIA), enabling soft marginal constraints via KL-regularization.

**GNN** - Graph neural network.

**GAT** - Graph attention network.

**GVAE** - Graph variational autoencoder.

**DGI** - Deep Graph Infomax.

**Cell-type permutation test** - A significance test that permutes cell labels to assess whether LR interactions depend on specific cell identities.

**Location permutation test** - A test that permutes spatial coordinates to evaluate whether LR interactions depend on spatial context.

**Spin test** - A spatial permutation test based on spherical rotation that preserves spatial autocorrelation, commonly used in cortical-analysis frameworks such as SCOTIA.
